# Population diversity analyses provide insights into key horticultural traits of Chinese native thymes

**DOI:** 10.1093/hr/uhac262

**Published:** 2022-08-02

**Authors:** Meiyu Sun, Yanan Zhang, Hongtong Bai, Guofeng Sun, Jinzheng Zhang, Lei Shi

**Affiliations:** Key Laboratory of Plant Resources, Institute of Botany, Chinese Academy of Sciences, Beijing 100093, China; China National Botanical Garden, Beijing 100093, China; Key Laboratory of Plant Resources, Institute of Botany, Chinese Academy of Sciences, Beijing 100093, China; China National Botanical Garden, Beijing 100093, China; University of Chinese Academy of Sciences, Beijing 100049, China; Key Laboratory of Plant Resources, Institute of Botany, Chinese Academy of Sciences, Beijing 100093, China; China National Botanical Garden, Beijing 100093, China; China National Botanical Garden, Beijing 100093, China; Key Laboratory of Plant Resources, Institute of Botany, Chinese Academy of Sciences, Beijing 100093, China; China National Botanical Garden, Beijing 100093, China; Key Laboratory of Plant Resources, Institute of Botany, Chinese Academy of Sciences, Beijing 100093, China; China National Botanical Garden, Beijing 100093, China

## Abstract

Chinese native thymes (CNTs) in the genus *Thymus* (family Lamiaceae) are rich in bioactive terpenes, which exert antiviral, anti-inflammatory, antioxidation, immunological, and antimicrobial effects. Plants exhibit morphological variation, including erect-type and creeping-type growth forms; however, the molecular mechanisms underlying important horticultural traits have not been determined. Here, we collected 39 CNTs providing strategic plant resources for studies of lignin, terpenoids, and glandular trichomes of thymes. Using resequencing data as well as phenotypic, metabonomic, phylogenetic, population genetic, and transcriptomic analyses, we identified and characterized key genes involved in lignin biosynthesis, terpenoid biosynthesis, and glandular trichome formation. We found many regulatory genes or transcription factors related to these three important horticultural traits, including genes encoding caffeic acid *O*-methyltransferase (COMT), terpene synthase (TPS), v-myb avian myeloblastosis viral oncogene homolog (MYB), and homeodomain-leucine zipper (HD-ZIP). Population diversity analyses provided insights into growth form, terpenoid, and glandular trichome evolution in CNTs. Furthermore, our results revealed that *T. mongolicus* accessions might be wild ancestors, and *T. quinquecostatus*, *T. quinquecostatus* var. *asiaticus*, and *T. quinquecostatus* var. *przewalskii* might be transitional accessions that derived from *T. mongolicus* accessions. Finally, *T. nervulosus*, *T. inaequalis*, *T. mandschuricus*, *T. curtus*, *T. amurensis*, *T. proximus*, *T. altaicus*, *T. roseus*, and *T. marschallianus* showed high divergence. We found evidence for introgression between erect-type European cultivated thymes and CNTs. These findings improve our understanding of the determinants of variation in horticultural traits and provide candidate loci for research and breeding.

## Introduction

*Thymus* (Lamiaceae family) is widely distributed and well known for its aromatic and pharmacological activity resulting from large amounts of bioactive terpenes in the leaves [1]. Fifteen species, two varieties, and one variant are recorded in the *Flora of China* [2] and local flora. However, the diversity of morphological and anatomical features of thyme species may be helpful in focusing on variation in horticultural traits. Several key horticultural traits, including the growth form (and lignin content), terpene content, and glandular trichomes, are not well characterized in the genus. With respect to growth form, thyme plants are erect-type or creeping-type, the prominent form. Erect-type thyme, which is tall and easy to harvest, is widely cultivated for pharmaceutical, food, and cosmetic applications in Europe and the USA, and has been recorded in the European Pharmacopoeia and US Pharmacopoeia [3]. Creeping-type thyme can form very strong root networks and plays a crucial ecological role by improving microbiological characteristics in the soil during decomposition in China [4]. The two growth forms may differ in lignin content in the stem. The lignin biosynthesis pathway has been evaluated in various plant taxa, including *Broussonetia papyrifera*, bamboo, and *Medicago polymorpha* [5–7]. For example, the caffeic acid *O*-methyltransferase (COMT) transcription factor plays an important role in this pathway [8–10]. However, the lignin biosynthesis pathway in thyme has not been determined, despite implications for the production of thyme. For example, increasing the lignin content in thyme stems to obtain relatively tall and upright plants can facilitate mechanized harvesting.

Chinese native thyme (CNT), known as ‘${\includegraphics{\bwartpath uhac262fx1}}$’ (Bailixiang), has large amounts of terpenes in the leaves. Terpenes have antiviral, antioxidation, anti-inflammatory, immunological, and antimicrobial effects, and can improve immunity, prevent thrombosis, relieve pain, and delay aging [11]. Thymol, γ-terpinene, and *p*-cymene have strong antioxidant, antimicrobial, and antifungal activities, and inhibit lactate production and decrease cellular glucose uptake [11–13]. α-Terpineol can reduce mechanical hypernociception and inflammatory responses, exhibits anticonvulsant activity, and exerts cardiovascular and gastroprotective effects [14]. 1,8-Cineole has well-established antiviral, anti-inflammatory, antioxidant, and antimicrobial functions [15]. Borneol has anti-inflammatory and antioxidant functions and increases blood–brain barrier permeability [16]. Germacrene-D is a sesquiterpene with insecticidal activity [17]. β-Caryophyllene is another sesquiterpene with several important pharmacological activities [18]. The terpenoid biosynthesis pathway derives from the methylerythritol phosphate pathway or mevalonate pathway in thyme [19]. Dimethylallyl diphosphate and isopentenyl diphosphate are both formed by transprenyltransferases to generate geranyl and farnesyl diphosphate. Terpene synthase (TPS) can catalyze geranyl and farnesyl diphosphate to form monoterpene and sesquiterpene. Terpenes are highly diverse plant secondary metabolites, and monoterpenes and sesquiterpenes are important components of medicines and fragrances and have roles in plant defense, emphasizing the importance of the functional verification of TPS genes in thyme.

Glandular trichomes are epidermal structures in some plants able to produce specialized multiple secondary metabolites. These secondary metabolites are frequently applied in the pharmaceutical and fragrance industries and contribute to plant adaptation [20]. The glandular trichome is the site of terpenoid storage and synthesis [21]. Glandular trichomes are widely found in Lamiaceae, including peltate and capitate trichomes [22]. Peltate trichomes have obvious subcuticular gaps and are the main organs for terpene synthesis [23]. Therefore, glandular trichomes are the prime targets for studying secondary metabolite synthesis and regulation in plants. Increasing the density of glandular trichomes is a common breeding strategy, emphasizing the importance of further understanding of the molecular basis of glandular trichome formation [20, 24]. The regulatory mechanisms underlying glandular trichome formation have been reported, including the vital roles of *R2R3-MYB*, *HD-ZIP IV*, *MYC1s*, *Glandular trichome-Specific WRKY 2* (*GSW2*), and *Transparent Testa Glabra 1* (*TTG1*) [22]. However, these genes have not been characterized in CNTs.

Here, we present a genomic variation map of 52 thyme accessions [39 CNTs and 13 European thymes (ETs)] of diverse origins via next-generation sequencing. By combining resequencing data, phenotypic data, metabonomics, phylogenetics, population genetics, and transcriptomics, we characterized three key horticultural traits (i.e. growth form, terpenoids, and glandular trichomes) in thyme. Our results will facilitate functional gene identification in thyme and provide a theoretical basis for molecular breeding.

## Results

### Growth form, terpenoids, and glandular trichomes in Chinese native thymes

**Figure 1:**
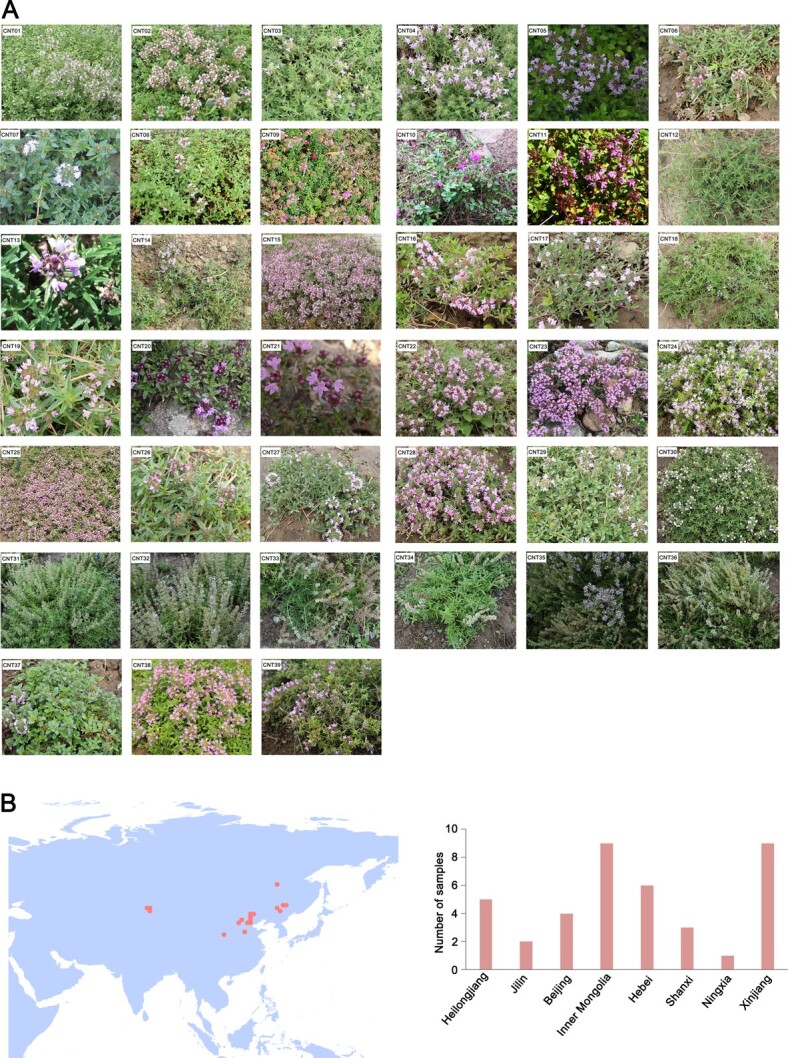
Collection of CNTs. (A) Images of CNTs. CNT01, *T. mandschuricus*; CNT02, *T. inaequalis*; CNT03, *T. curtus*; CNT04, *T. amurensis*; CNT05, *T. inaequalis*; CNT06, *T. quinquecostatus* var. *asiaticus*; CNT07, *T. nervulosus*; CNT08, *T. mongolicus*; CNT09, *T. mongolicus*; CNT10, *T. mongolicus*; CNT11, *T. mongolicus*; CNT12, *T. quinquecostatus* var. *asiaticus*; CNT13, *T. quinquecostatus* var. *asiaticus*. CNT14, *T. quinquecostatus* var. *przewalskii*; CNT15, *T. quinquecostatus* var. *asiaticus*; CNT16, *T. quinquecostatus* var. *asiaticus*; CNT17, *T. quinquecostatus*; CNT18, *T. quinquecostatus*; CNT19, *T. quinquecostatus* var. *asiaticus*; CNT20, *T. quinquecostatus*; CNT21, *T. quinquecostatus*; CNT22, *T. mongolicus*; CNT23, *T. quinquecostatus*; CNT24, *T. quinquecostatus* var. *przewalskii*; CNT25, *T. quinquecostatus* var. *przewalskii*; CNT26, *T. quinquecostatus* var. *asiaticus*; CNT27, *T. quinquecostatus* var. *przewalskii*; CNT28, *T. quinquecostatus* var. *przewalskii*; CNT29, *T. mongolicus*; CNT30, *T. roseus*; CNT31, *T. marschallianus*; CNT32, *T. marschallianus*; CNT33, *T. marschallianus*; CNT34, *T. marschallianus*; CNT35, *T. marschallianus*; CNT36, *T. marschallianus*; CNT37, *T. proximus*; CNT38, *T. altaicus*; CNT39, *T. quinquecostatus*. CNT01–CNT30 and CNT37–CNT39 are creeping-type (CNTC) thymes. CNT31–CNT36 are semicreeping-type. (B) Geographical distribution and collection of collected CNTs in China.

We collected 39 CNT accessions assigned to the following 11 species and 2 varieties based on morphological characteristics: *T. quinquecostatus*, *T. quinquecostatus* var. *przewalskii*, *T. quinquecostatus* var. *asiaticus*, *T. mongolicus*, *T. altaicus*, *T. amurensis*, *T. curtus*, *T. inaequalis*, *T. mandschuricus*, *T. marschallianus*, *T. nervulosus*, *T. proximus*, and *T. roseus* (Fig. 1A and Supplementary Data Table S1). All samples were collected at sites west of the Yellow River in China, including Heilongjiang Province, Jilin Province, Inner Mongolia Autonomous Region, Beijing Municipal, Hebei Province, Shanxi Province, Xinjiang Uygur Autonomous Region, and Ningxia Hui Autonomous Region (Fig. 1B and Supplementary Data Table S1). The most thyme accessions were obtained from the Xinjiang Uygur Autonomous Region and the Inner Mongolia Autonomous Region (nine CNT accessions each).

CNT and ET show two main growth forms, erect-type and creeping-type, which may be related to the lignin content in their stems. *T. marschallianus* accessions (CNT31–CNT36) are semicreeping-type, and the remaining CNT accessions (CNT01–CNT30 and CNT37–CNT39) are creeping-type (CNTC, Fig. 1A). Six ETs (ET08–ET13), *T. longicaulis*, *T. comosus*, *T. guberlinesis*, *Thymus pulegioides* ‘Golden Dwarf’, *Thymus serpyllum* ‘Aureus’, and *T. praecox* Opiz subsp. *polytrichus* (A. Kern. ex Borbs) *Jalas*, are creeping-type (ETC). Seven ETs (ET01–ET07), *T. vulgaris* ‘Compactus’, *T. vulgaris* ‘Elsbeth’, *T. vulgaris* ‘Fleur Provenule’, *T. vulgaris* ‘Fragrantissimus’, *T. vulgaris* ‘Pink Selection’, *Thymus thracicus*, and *T. rotundifolia*, are erect-type (ETE) (Supplementary Data Fig. S1). We created new germplasm resources by hybridizing European erect-type thyme with Chinese creeping-type thyme. Interestingly, in the *F*_1_ generations of six populations, the erect and creeping traits exhibited maternal inheritance (Supplementary Data Fig. S2). The *F*_1_ lines were erect or semierect when the female parent was an erect-type thyme, and creeping or semicreeping when the female parent was a creeping-type thyme. These *F*_1_ populations can self-cross to generate *F*_2_ populations, providing the foundation for quantitative trait locus (QTL) mapping and gene function verification, and a basis for improving mechanized harvesting in agricultural production.

**Figure 2:**
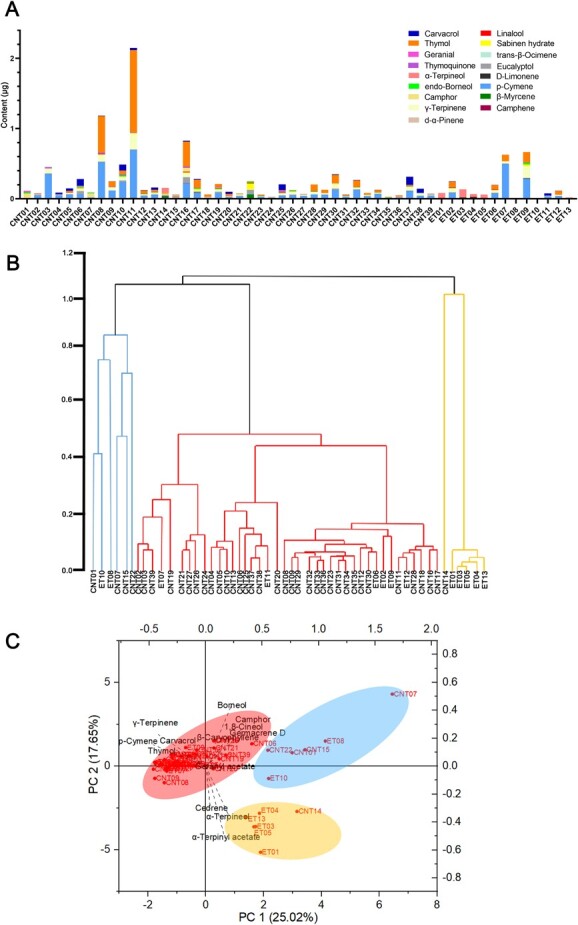
Leaf volatile terpenoid compositions of 52 thyme accessions. (A) Contents of main volatile terpenoids in leaves. (B) Cluster analysis of 52 thymes based on the volatile terpenoid compositions. (C) PCA of thymes from different regions based on volatile terpenoid compositions.

As determined by gas chromatography–mass spectrometry (GC–MS), the terpene profiles differed substantially among the leaves of 52 thyme accessions. Of 55 terpenes identified, 28 were monoterpenes and 16 were sesquiterpenes (Supplementary Data Table S2). *p*-Cymene, γ-terpinene, and thymol were the most abundant and the most common terpenes in most samples (other than CNT01, CNT07, CNT15, ET03, ET05, ET08, and ET10) (Fig. 2A and Supplementary Data Table S2). We performed a cluster analysis to evaluate similarities and relationships among *Thymus* species based on their major components and constructed a dendrogram. The samples were assigned to three clusters (Fig. 2B). Six samples were assigned to the first cluster, all of which were creeping-type. The second cluster included samples rich in thymol and carvacrol. The third cluster consisted of CNT14 and five ETs, all characterized by high quantities of α-terpinyl acetate and α-terpineol (Supplementary Data Table S2). In a principal component analysis (PCA) (Fig. 2C), the first two main components explained 42.67% of the total variance in volatile profiles. The first principal component explained 25.02% of the variance and showed positive correlations with camphor (0.35), germacrene-D (0.33), and 1,8-cineol (0.32). The second component explained 17.65% of the variance and exhibited positive correlations with *p*-cymene (0.15), carvacrol (0.13), and thymol (0.12). The PCA results were similar to those of the cluster analysis (Fig. 2B and C). Three CNT accessions with high thymol contents, *T. mongolicus* (CNT08), *T. mongolicus* (CNT11), and *T. quinquecostatus* var. *asiaticus* (CNT16), are promising for agricultural production and medical applications based on terpene components (Fig. 2A).

**Figure 3:**
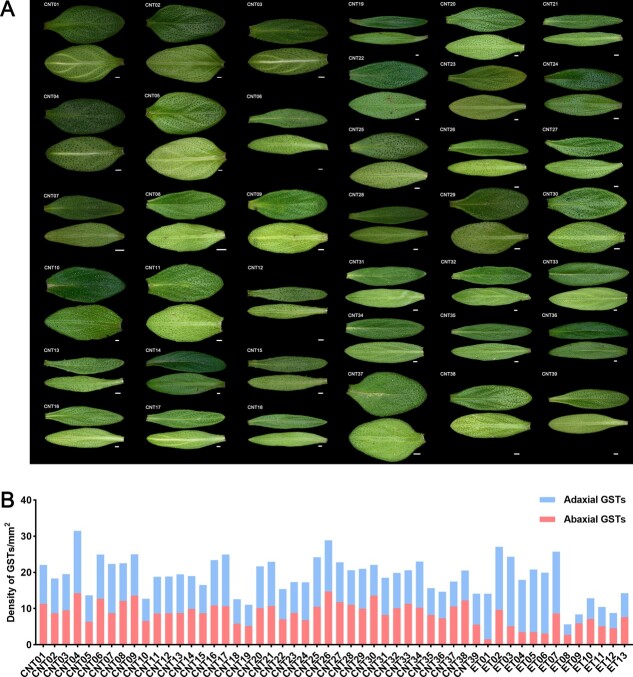
Adaxial and abaxial leaves images and glandular trichome densities in thymes. (A) Adaxial and abaxial leaves images of 39 CNTs. (B) Density of glandular trichomes on adaxial and abaxial leaves in 52 thymes.

The well-known Chinese name ‘${\includegraphics{\bwartpath uhac262fx2}}$’ (Bailixiang) for thyme reflects its strong fragrance, which is detectable over a great distance due to large amounts of aromatic ingredients, namely terpenes. Leaf shape and color varied substantially among samples. Glandular trichome distributions showed macroscopic variation (Supplementary Data Fig. S3). Glandular trichomes on the abaxial or adaxial surface were abundant and showed similar patterns of variation. However, in ETE samples there were significantly more adaxial glandular trichomes than abaxial glandular trichomes and more total glandular trichomes than the number in ETC. The total glandular trichome density in leaves was 5.61–31.46 per mm^2^ in CNTs and ETs (Fig. 3 and Supplementary Data Table S3). The leaf area ranged from 5.63 mm^2^ to 35.25 mm^2^. These analyses revealed substantial variation in key horticultural traits, including growth form, leaf terpenoid composition and content, and leaf glandular trichome density, among 52 thyme accessions.

### Population diversity analysis of thymes

Divergence and relationships among thyme accessions were examined by resequencing of 39 CNTs and 13 ETs (cultivated and wild thymes). A total of 1.23 billion paired-end reads (369.37 Gb) were obtained. The average depth was 9× (Supplementary Data Tables S4–S6). Insertion–deletions (InDels) and single-nucleotide polymorphisms (SNPs) were identified and analyzed in 52 thyme accessions (Supplementary Data Tables S7–S9, Supplementary Data Figs S4 and S5). On average, there were 527 194 SNPs, 10 150 coding sequence insertions, and 7046 coding sequence deletions in 52 thyme accessions (Supplementary Data Tables S7 and S8). We detected 7978–16 854 genes with non-synonymous SNPs and 3008–7511 genes with InDels (Supplementary Data Table S9).

**Figure 4:**
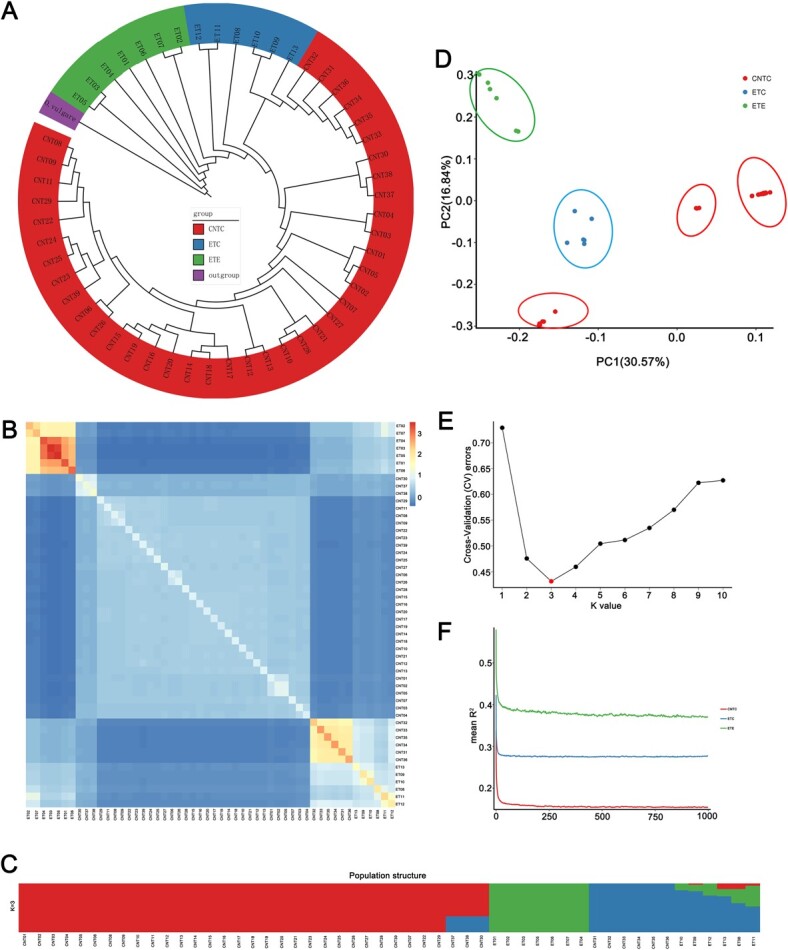
Genetic diversity, population structure, and evolution of thymes. (A) Phylogenetic tree of CNTs and ETs. Red, creeping-type or semicreeping-type CNT (CNCT); blue, creeping-type European thyme (ETC); green, erect-type European thyme (ETE); purple, outgroup, *Origanum vulgare* (Chinese wild oregano, diverged ~1.47 Mya). Ecological factors for wild thyme populations native to China are shown in Supplementary Data Table S1. (B) Heat map of the genetic relationship between two individuals in the thyme populations. (C) Structure of the thyme populations (*K* = 3). Red, CNTs, *T. quinquecostatus*, *T. quinquecostatus* var. *asiaticus*, *T. quinquecostatus* var. *przewalskii*, *T. mongolicus*, *T. amurensis*, *T. curtus*, *T. inaequalis*, *T. marschallianus*, and *T. nervulosus*; red + blue, three accessions of *T. roseus* (CNT30), *T. proximus* (CNT37), and *T. altaicus* (CNT38); green, ETE; blue, six accessions of *T. marschallianus* (CNT31–CNT36); blue + green (+ red), ETC. (D) PCA of the thyme populations. PC1 and PC2 split the thyme populations into five clusters. (E) Cross-validation errors for the population structure analysis. The *x*-axis represents the *K* value, while the *y*-axis indicates the cross-validation error. The red dot shows *K* = 3 with the lowest cross-validation error. (F) LD decay within different subgroups of CNTs and ETs. LD (*y*-axis) decays as a function of the genomic distance between polymorphisms (*x*-axis) in CNTC (solid red line), ETC (solid blue line), and ETE (solid green line) thyme populations. LD was measured as *R*^2^.

As shown in Fig. 4A, phylogenetic trees were generated with *Origanum vulgare* (Sun *et al.*, unpublished data, Chinese wild oregano, genome size ~641.87 Mb, divergence date ~1.47 Mya) as the outgroup. Molecular dating using *Sorghum bicolor* for fossil calibration indicated that *Origanum*–*Thymus* diverged ~7.47 Mya (Sun *et al.*, unpublished data)*.* The phylogenetic analyses revealed that the *T. mongolicus* accessions might be wild ancient accessions, with the subsequent divergence of *T. quinquecostatus*, *T. quinquecostatus* var. *przewalskii*, *T. quinquecostatus* var. *asiaticus*. *T. nervulosus*, *T. inaequalis*, *T. mandschuricus*, *T. curtus*, *T. amurensis*, *T. proximus*, *T. altaicus*, *T. roseus*, and *T. marschallianus* diverged more recently.

PCA based on the SNP dataset was used to study the differentiation and relationships among different thyme accessions. Five clusters were consistently detected (Fig. 4D). The first two principal components accounted for 30.57% and 16.84% of the total variance, respectively. To further analyze the genetic relationships between these thyme accessions, we performed a structure analysis using ADMIXTURE. At a *K* value of 1–10, 39 CNTs and 13 ETs had high diversity (Supplementary Data Fig. S6). At a *K*-value of 3 (Fig. 4E), CNTC, ETC, and ETE could be readily distinguished (Fig. 4C), in coincidence with the PCA results (Fig. 4D) and the analysis of pairwise genetic relationships between individuals (Fig. 4B). We found that 39 CNT accessions could be clustered into three independent groups, consistent with the morphology-based classical taxonomy. A well-clustered group included *T. mongolicus*, *T. quinquecostatus*, *T. quinquecostatus* var. *przewalskii*, *T. quinquecostatus* var. *asiaticus*, *T. amurensis*, *T. curtus*, *T. inaequalis*, *T. marschallianus*, *T. nervulosus*, *T. roseus*, *T. proximus*, and *T. altaicus*, all of which were creeping-type thymes. Nevertheless, *T. marschallianus* (CNT31–CNT36) accessions clustered with ETs, which were semicreeping-type thymes.

**Figure 5:**
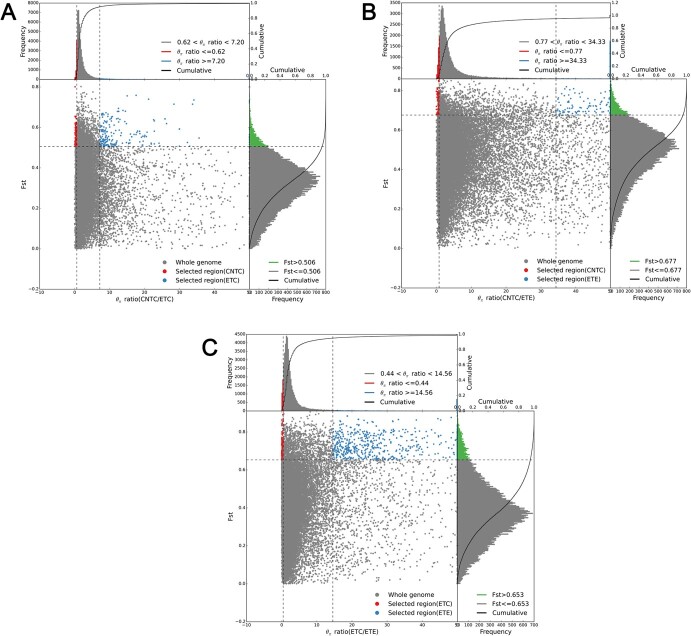
*F*_ST_- and θπ-based selective sweep identification in thymes. (A) *F*_ST_- and θπ-based selective sweep identification between CNTC and ETC. (B) *F*_ST_- and θπ-based selective sweep identification between CNTC and ETE. (C) *F*_ST_- and θπ-based selective sweep identification between ETC and ETE.

We further researched population-level heterozygosity in the three groups. We found that population heterozygosity in ETC (15.38%) was significantly higher than that in CNTC (13.08%) and ETE (13.12%) (both *P* < .05). The linkage disequilibrium (LD) decay values, calculated based on the squared correlation coefficient (*R*^2^) in three populations, revealed substantial differences among the CNTC, ETC, and ETE populations (Fig. 4F). The ranking of these three populations for LD decay rate from low to high was as follows: ETE, ETC, and CNTC. Generally, the higher the degree of domestication and the higher the intensity of selection, the slower the rate of LD decay. This also indicates that the erect-type thyme was formed by the continuous domestication of the creeping-type wild thyme.

### Adaptive evolution of Chinese native thymes associated with key horticultural traits

Key horticultural traits, such as growth form (Fig. 1A), leaf terpene compounds (Fig. 2), and leaf glandular trichome density (Fig. 3), clearly differed among the 52 accessions. To investigate the role of selection during varietal inheritance and improvement in thymes, candidate genes and regions under selection were separately identified for CNTC versus ETC, CNTC versus ETE, and ETC versus ETE based on growth form. Selective sweeps identified using fixation statistics (*F*_ST_) revealed 1134 genes under selection (263 selected regions) from CNTC to ETC (Fig. 5A), 1691 genes under selection (288 selected regions) from CNTC to ETE (Fig. 5B), and 936 genes under selection (195 selected regions) from ETC to ETE (Fig. 5C). We found that 53 key genes related to lignin biosynthesis (one *4CL*, one *C4H*, two *CCoAOMT*s, twelve *CCR*s, five *HCT*s, and three *PER/LAC*s), terpenoid biosynthesis (five *CYP*s and one *TPS*), and glandular trichome formation (two *ARF3*s, one *CyCB2*, six *HD-ZIP*s, two *IAA15*s, six *MYC1*s, five *MYB*s, and one *TTG1*) were under selection between CNTC and ETC (Supplementary Data Table S10); 45 key genes related to lignin biosynthesis (one *4CL*, one *C3H*, one *CCR*, and three *HCT*s), terpenoid biosynthesis (five *CYP*s, one *MDS*, and two *TPS*s), and glandular trichome formation (four *ARF3*s, two *CyCB2*s, one *GIS*, four *GSW2*s, five *HD-ZIP*s, two *IAA15*s, five *MYC1*s, seven *MYB*s, and one *TTG1*) were under selection between CNTC and ETE (Supplementary Data Table S11); and 31 key genes related to lignin biosynthesis (two *4CL*s and four *HCT*s), terpenoid biosynthesis (seven *CYP*s, one *HMGS*, and one *TPS*), and glandular trichome formation (two *GSW2*s, three *HD-ZIP*s, four *MYC1*s, six *MYB*s, and one *TTG1*) were under selection between ETC and ETE (Supplementary Data Table S12).

The selective sweeps identified based on nucleotide diversity (θπ) revealed 703 genes under selection (244 selected regions) from CNTC to ETC (Fig. 5A), 699 genes under selection (225 selected regions) from CNTC to ETE (Fig. 5B), and 978 genes under selection (218 selected regions) from ETC to ETE (Fig. 5C). We also found that 23 key genes related to lignin biosynthesis (one *CAD/SAD* and five *HCT*s), terpenoid biosynthesis (five *CYP*s and four *TPS*s), and glandular trichome formation (three *HD-ZIP*s, one *MYC1*, and four *MYB*s) were under selection between CNTC and ETC (Supplementary Data Table S13); 16 key genes related to lignin biosynthesis (two *CAD/SAD*s and one *HCT*), terpenoid biosynthesis (four *CYP*s), and glandular trichome formation (one *GSW2*, four *MYC1*s, three *MYB*s, and one *TTG1*) were under selection between CNTC and ETE (Supplementary Data Table S14); and 28 key genes related to lignin biosynthesis (one *CAD/SAD*, one *HCT*, and one *PER/LAC*), terpenoid biosynthesis (10 *CYP*s), and glandular trichome formation (one *GSW2*, one *HD-ZIP*, one *IAA15*, five *MYC1*s, six *MYB*s, and one *TTG1*) were under selection between ETC and ETE (Supplementary Data Table S15).

**Figure 6:**
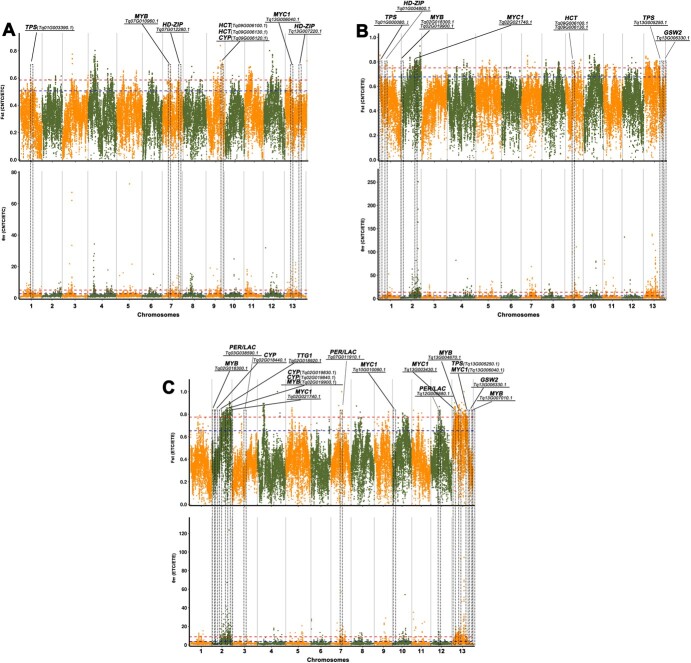
Selective sweeps and identification of candidate genes underlying key horticultural traits in thymes. (A) Among genes showing the signature of selection between CNTC and ETC, *HCT*s are related to lignin biosynthesis; *TPS* and *CYP* are related to terpenoid biosynthesis; and *HD-ZIP*, *MYB*, and *MYC1* are related to glandular trichome formation. (B) Among genes showing the signature of selection between CNTC and ETE, *HCT*s are related to lignin biosynthesis, and *TPSs* are related to terpenoid biosynthesis; *HD-ZIP*, *MYB*, *MYC1*, and *GSW2* are related to glandular trichome formation. (C) Among genes showing the signature of selection between ETE and ETC, *PER/LAC*s are related to lignin biosynthesis; *TPS* and *CYP*s are related to terpenoid biosynthesis; and *MYB*s, *MYC1*s, *GSW2*, and *TTG1* are related to glandular trichome formation. Manhattan plots using the randomly downsampled SNP set for all linkage groups. Manhattan plots show the detection of selection based on both *F*_ST_ and θπ (from top to bottom). The locations of orthologous genes associated with key horticultural traits are indicated in the Manhattan plots. Red and blue dashed lines indicate the 1% and 5% thresholds, respectively. CNTC, creeping-type Chinese native thyme; ETC, creeping-type European thyme; ETE, erect-type European thyme; SNP, single-nucleotide polymorphism; HD-ZIP, homeodomain-leucine zipper.

Analyses based on both *F*_ST_ and θπ revealed many key genes under selection (Fig. 6). We found that many key genes related to lignin biosynthesis (such as the *HCT* genes *Tq09G006100.1* and *Tq09G006130.1*), terpenoid biosynthesis (such as the TPS-encoding gene *Tq01G003390.1* and CYP-encoding gene *Tq09G006120.1*), and glandular trichome formation [such as the homeodomain-leucine zipper (1)-encoding genes *Tq07G012280.1* and *Tq13G007220.1*, MYB-encoding gene *Tq07G010960.1*, and *MYC1* gene *Tq13G006040.1*] were under selection between CNTC and ETC (Fig. 6A). Many key genes related to lignin biosynthesis (such as the *HCT* genes *Tq09G006100.1* and *Tq09G006130.1*), terpenoid biosynthesis (such as the TPS-encoding genes *Tq01G000080.1* and *Tq13G005250.1*), glandular trichome formation (such as the HD-ZIP-encoding gene *Tq01G004800.1*, MYB-encoding genes *Tq02G018300.1* and *Tq02G019900.1*, *MYC1* gene *Tq02G021740.1*, and *GSW2* gene *Tq13G006330.1*) were also under selection between CNTC and ETE (Fig. 6B). Many key genes related to lignin biosynthesis (such as the *PER/LAC* genes *Tq03G038590.1*, *Tq07G011910.1*, and *Tq12G006680.1*), terpenoid biosynthesis (such as the TPS-encoding gene *Tq13G005250.1* and CYP-encoding genes *Tq02G018440.1*, *Tq02G019830.1*, and *Tq02G019840.1*), and glandular trichome formation (such as the *GSW2* gene *Tq13G006330.1*; *MYC1* genes *Tq02G021740.1*, *Tq10G010090.1*, *Tq13G003430.1*, and *Tq13G006040.1*; MYB-encoding genes *Tq02G018300.1*, *Tq02G019900.1*, *Tq13G004670.1*, and *Tq13G007010.1*; and *TTG1* gene *Tq02G018920.1*) were under selection between ETC and ETE (Fig. 6C). The key genes related to the three important horticultural traits provide a foundation for functional assays.

GO (Gene Ontology), COG (Clusters of Orthologous Groups), and KEGG (Kyoto Encyclopedia of Genes and Genomes) analyses of the candidate genes involved in lignin, terpenoid, and trichome traits were performed to evaluate functional enrichment (Supplementary Data Figs S6–S8). A KEGG enrichment analysis indicated that most of the candidate gene families in the CNTC–ETC comparison were involved in phenylpropanoid biosynthesis, stilbenoid, plant–pathogen interaction, RNA polymerase, flavonoid biosynthesis, diarylheptanoid and gingerol biosynthesis, carbon metabolism, pentose and glucuronate interconversions, citrate cycle (TCA cycle) and protein processing in the endoplasmic reticulum (Supplementary Data Fig. S7). In the CNTC–ETE comparison, candidate gene families were involved in plant hormone signal transduction, glutathione metabolism, plant–pathogen interaction, galactose metabolism, starch and sucrose metabolism, RNA degradation, inositol phosphate metabolism, biosynthesis of amino acids, glycolysis/gluconeogenesis, purine metabolism, pyrimidine metabolism, cutin and wax biosynthesis, diterpenoid biosynthesis, and phenylpropanoid biosynthesis (Supplementary Data Fig. S8). In the ETC–ETE comparison, candidate gene families were clustered in diterpenoid biosynthesis, spliceosome, plant–pathogen interaction, plant hormone signal transduction, protein processing in endoplasmic reticulum, cysteine and methionine metabolism, starch and sucrose metabolism, carotenoid biosynthesis, purine metabolism, phenylalanine metabolism, and glycerophospholipid metabolism (Supplementary Data Fig. S9). These metabolic processes might be related to the thyme lignin and terpenoid contents, flower color, and glandular trichomes.

### Phylogenetic analysis of genes encoding COMT, TPS, HD-ZIP, and MYB

**Figure 7:**
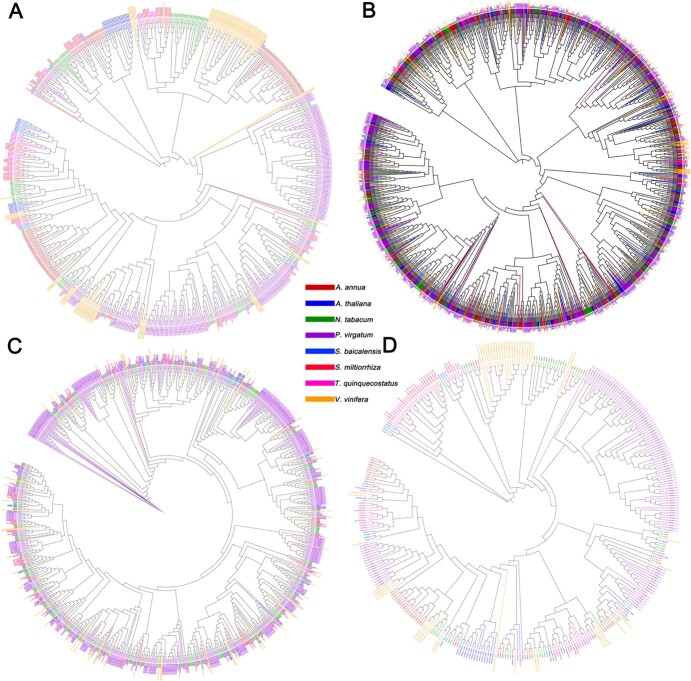
Phylogenetic analysis of *T. quinquecostatus* (CNT39) and related species based on genes encoding COMT, TPS, MYB, and HD-ZIP. (A) Phylogeny based on genes encoding COMT in *T. quinquecostatus*, *A. annua*, *A. thaliana*, *N. tabacum*, *P. virgatum*, *S. baicalensis*, *S. miltiorrhiza*, and *V. vinifera*. (B) Phylogenetic analysis based on genes encoding TPS in *T. quinquecostatus* and related species. (C) Phylogenetic analysis based on genes encoding MYB in *T. quinquecostatus* and related species. (D) Phylogenetic analysis based on genes encoding HD-ZIP in *T. quinquecostatus* and related species.

By an extensive review of the literature [8–10, 19–22], we found that many studies of the three important horticultural traits have focused on genes encoding COMT, TPS, HD-ZIP, and MYB. The chromosome-level genome of *T. quinquecostatus* (CNT39, an important wild thyme species in China) has been assembled and annotated by our research team, providing a basis for analyses of these genes [22]. Caffeic acid *O*-methyltransferase (COMT) is a key transcription factor involved in lignin biosynthesis and growth form. A sequence similarity search revealed 37, 22, 55, 74, 496, 110, 122, and 112 COMT-encoding genes in *T. quinquecostatus*, *Arabidopsis thaliana*, *Artemisia annua*, *Nicotiana tabacum*, *Panicum virgatum*, *Salvia miltiorrhiza*, *Scutellaria baicalensis*, and *Vitis vinifera*, respectively (Fig. 7A). TPS-encoding genes are involved in terpenoid biosynthesis. We found that these important genes physically clustered on thyme pseudochromosomes. We identified 69, 38, 131, 99, 197, 87, 45, and 98 TPS-encoding genes in the aforementioned eight genomes, respectively (Fig. 7B). MYB and HD-ZIP are the two most important classes of transcription factors involved in trichome development. We identified 175, 239, 326, 421, 512, 191, 185, and 177 MYB-encoding genes (Fig. 7C), as well as 87, 85, 100, 216, 382, 83, 98, and 64 HD-ZIP-encoding genes, in the aforementioned eight genomes, respectively (Fig. 7D). A phylogenetic analysis of genes encoding COMT, TPS, HD-ZIP, and MYB in these eight sequenced plant genomes indicated multiple recent species-specific tandem duplication events.

**Figure 8:**
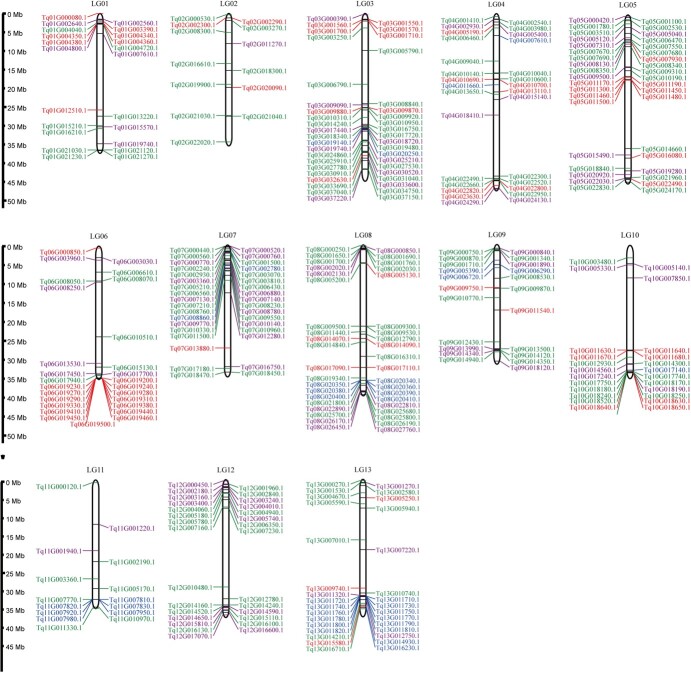
Chromosomal distribution of genes encoding COMT, TPS, MYB, and HD-ZIP in *T. quinquecostatus* (CNT39). Genes are shown beside the chromosomes with the corresponding *T. quinquecostatus* locus IDs in parentheses based on MCScanX results. A total of 37 genes encoding COMT (blue), 68 genes encoding TPS (red), 175 genes encoding MYB (green), and 87 genes encoding HD-ZIP (purple) had tandem duplication events in 13 chromosomes of *T. quinquecostatu*s (Supplementary Data Table S16).

By a bioinformatics analysis of *T. quinquecostatus* (CNT39), we identified tandem duplication events in 37 genes encoding COMT, 68 genes encoding TPS, 175 genes encoding MYB, and 87 genes encoding HD-ZIP on 13 chromosomes (Fig. 8 and Supplementary Data Table S16)*.* We identified 25, 49, 29, and 10 tandem duplication events in genes encoding COMT, TPS, MYB, and HD-ZIP, respectively (Supplementary Data Table S16). COMT-encoding genes showed up to 12 tandem duplications on chromosome 13, TPS-encoding genes showed up to 14 tandem duplications on chromosome 6, MYB-encoding genes showed eight tandem duplications on chromosome 10, and HD-ZIP-encoding genes showed four tandem duplications on chromosome 7 (Fig. 8). The identification of these genes in *T. quinquecostatus* provides important biological information for studies of key horticultural traits, such as the growth form, terpenoids, and glandular trichome content, in CNTs.

### RNA-seq analysis of the mechanism underlying lignin biosynthesis and modular organization

**Figure 9:**
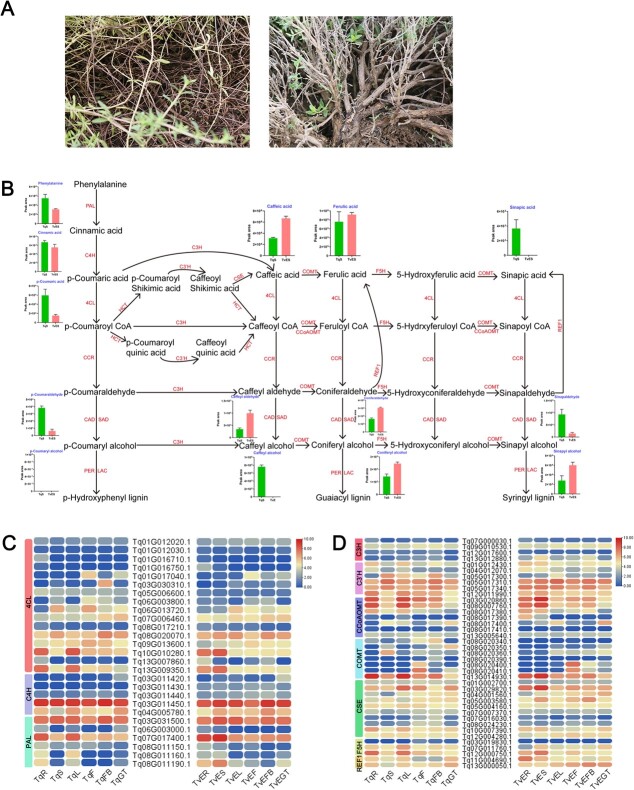
Schematic overview of lignin biosynthesis in thyme. (A) Stem images of *T. quinquecostatus* and *T. vulgaris* ‘Elsbeth’. (B) Lignin biosynthesis pathway. PAL, phenylalanine ammonia lyase; C4H, cinnamate 4-hydroxylase; 4CL, 4-coumarate-CoA ligase; CCR, cinnamoyl-CoA reductase; CAD/SAD, cinnamyl/sinapyl alcohol dehydrogenase; PER, peroxidase; LAC, laccase; C3H, coumarate 3-hydroxylase; HCT, hydroxycinnamoyl-CoA shikimate/quinate hydroxycinnamoyl transferase; C3′H, coumaroyl shikimate/quinate 3′-hydroxylase; COMT, caffeic acid *O*-methyltransferase; CCoAOMT, caffeoyl-CoA *O*-methyltransferase; F5H, ferulate 5-hydroxylase; CSE, caffeoyl shikimate esterase; REF1, coniferyl-aldehyde dehydrogenase. (C) Expression heat map of DEGs encoding PAL, C4H, and 4CL. (D) Expression heat map of DEGs encoding C3H, COMT, F5H, C3′H, CSE, CCoAOMT, and REF1. TqR, *T. quinquecostatus* root; TqS, *T. quinquecostatus* stem; TqL, *T. quinquecostatus* leaf; TqF, *T. quinquecostatus* flower; TqFB, *T. quinquecostatus* flower at the bud stage; TqGT, *T. quinquecostatus* glandular trichome; TvER, *T. vulgaris* ‘Elsbeth’ root; TvES, *T. vulgaris* ‘Elsbeth’ stem; TvEL, *T. vulgaris* ‘Elsbeth’ leaf; TvEF, *T. vulgaris* ‘Elsbeth’ flower; TvEFB, *T. vulgaris* ‘Elsbeth’ flower at the bud stage; TvEGT, *T. vulgaris* ‘Elsbeth’ glandular trichome.

We have previously analyzed the mechanism underlying terpenoid biosynthesis and glandular secretory trichome formation based on transcriptome data for *T. vulgaris* ‘Elsbeth’ and *T. quinquecostatus* [22]. In this study, we focus on lignin biosynthesis using the previously generated transcriptome data. The lignin contents of the stem differed substantially between *T. vulgaris* ‘Elsbeth’ (erect-type) and *T. quinquecostatus* (creeping-type) (Fig. 9). Contents of intermediate metabolites related to lignin biosynthesis in *T. quinquecostatus* and *T. vulgaris* ‘Elsbeth’ are shown in Fig. 9B. Phenylalanine, *p*-coumaric acid, and cinnamic acid contents were higher in *T. quinquecostatus* than in *T. vulgaris* ‘Elsbeth’. In contrast, the caffeic acid content was lower in *T. quinquecostatus* than in *T. vulgaris* ‘Elsbeth’. Surprisingly, 14 key gene families (*PAL*, *C4H*, *4CL*, *C3H*, *COMT*, *F5H*, *C3′H*, *CSE*, *CCoAOMT*, *REF1*, *CCR*, *CAD/SAD*, *PER/LAC*, and *HCT*) in the lignin biosynthesis pathway were differentially expressed between species (Fig. 9B). In lignin biosynthesis, phenylalanine ammonia lyase (PAL) is the first vital rate-limiting enzyme, and the related gene family included six differentially expressed genes (DEGs) (Fig. 9C). A heat map of DEGs showed that 5 *C4H*, 16 *4CL*, 32 *CCR*, 43 *CAD/SAD*, 62 *PER/LAC*, 4 *C3H*, 7 *COMT*, 3 *F5H*, 6 *C3′H*, 10 *CSE*, 7 *CCoAOMT*, 59 *HCT*, and 2 *REF1* genes were related to lignin biosynthesis (Fig. 9C and Supplementary Data Fig. S10). These results suggested that the concentrations of intermediate metabolites related to lignin biosynthesis in *T. vulgaris* ‘Elsbeth’ stem were higher than in *T. quinquecostatus*, which may explain why *T. vulgaris* ‘Elsbeth’ was erect and *T. quinquecostatus* was creeping.

**Figure 10:**
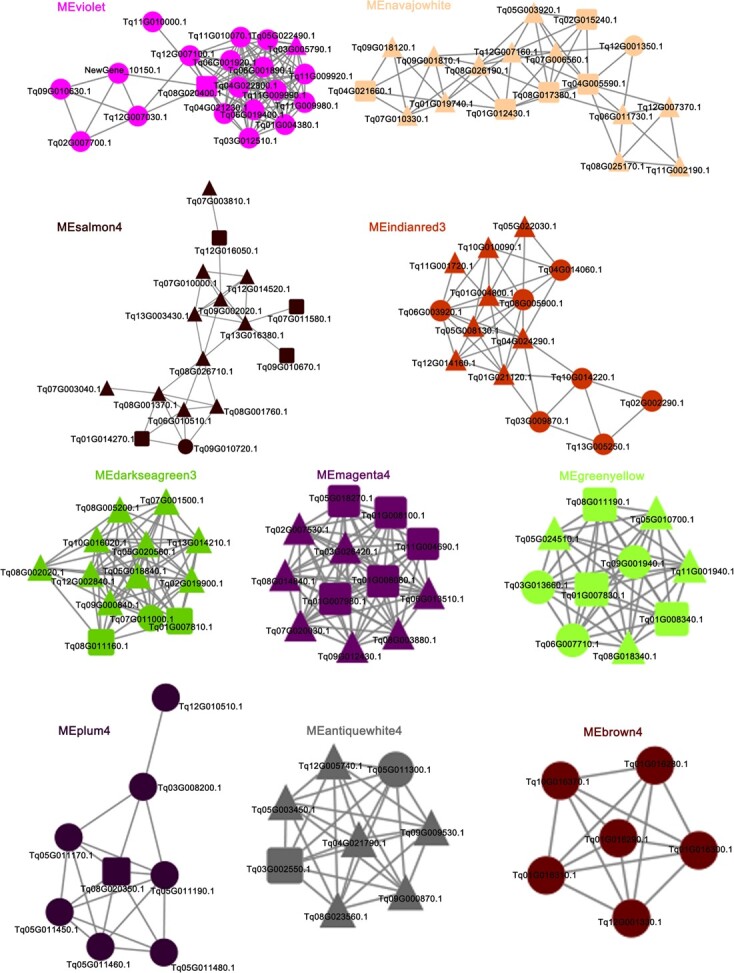
Coexpression networks of genes related to lignin biosynthesis, terpenoid biosynthesis, and glandular trichome formation in thyme. A total of 22 lignin-, 45 terpenoid-, and 59 trichome-related genes in 10 networks (Supplementary Data Table S17). Squares, key genes in lignin biosynthesis; circles, key genes in terpenoid biosynthesis; triangles, key genes in glandular trichome formation.

A weighted gene coexpression network has been generated for *T. vulgaris* ‘Elsbeth’ and *T. quinquecostatus* samples [22]. To further study the molecular basis of lignin, terpenoid, and glandular trichome traits, hub genes were identified. We identified 126 coexpressed genes related to lignin and terpenoid biosynthesis and trichome formation in 10 networks (Fig. 10). Among the 10 networks, *CAD/SAD*, *CCR*, and *HCT* were related to lignin biosynthesis, hub genes encoding TPS and CYP were related to terpenoid biosynthesis, and hub genes encoding MYB and HD-ZIP along with *GSW2*, *MYC1*, and *CyCB2* were associated with glandular trichome formation and displayed a high edge number (Supplementary Data Table S17). The coexpression networks provide further support for the selection of candidate genes related to key horticultural traits in thymes.

### Validation of expression of key genes and functional characterization of recombinant γ-terpinene synthase TqTPS2

**Figure 11:**
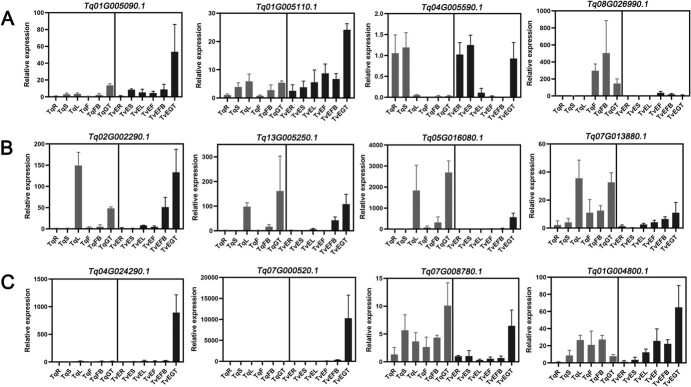
Gene expression patterns for four genes in each pathway as verified by qRT–PCR. (A) Relative expression levels of four *PER/LAC* genes related to lignin biosynthesis. (B) Relative expression levels of *TPS*s in terpenoid biosynthesis. (C) Relative expression levels of *HD-ZIP*s involved in glandular trichome formation. Gray bars represent relative expression levels in the root, stem, leaf, flower, flower bud, and glandular trichome in *T. quinquecostatus*. Black bars represent relative expression levels in the root, stem, leaf, flower, flower bud, and glandular trichome in *T. vulgaris* ‘Elsbeth’. Error bars represent the standard deviation.

To confirm the reliability of our transcriptome data, we selected four genes in each pathway to verify the expression levels by quantitative real-time PCR (qRT–PCR). The expression patterns of these genes were generally in agreement with the transcriptome data. The relative expression levels of *Tq13G005250.1* were higher in the *T. quinquecostatus* glandular trichome (TqGT) and *T. vulgaris* ‘Elsbeth’ glandular trichome (TvEGT) than in other tissues (Fig. 11A). The expressions of another three genes in the glandular trichome were relatively high. These results indicated that terpenoids are synthesized and stored in glandular trichomes [22]. The relative expression levels of *Tq01G004800.1* in the leaf, flower, flower bud, and glandular trichome were high in both *T. quinquecostatus* and *T. vulgaris* ‘Elsbeth’ (Fig. 11B). The expression levels of *Tq04G005590.1* in the root and stem were higher than those in other tissues, indicating that it may contribute to lignin biosynthesis (Fig. 11C). The gene coexpression network related to terpenoids has been analyzed in our previous study, in which *Tq13G005250.1* clustered with previously reported γ-terpinene synthase-encoding genes in oregano and thyme [22].

**Figure 12:**
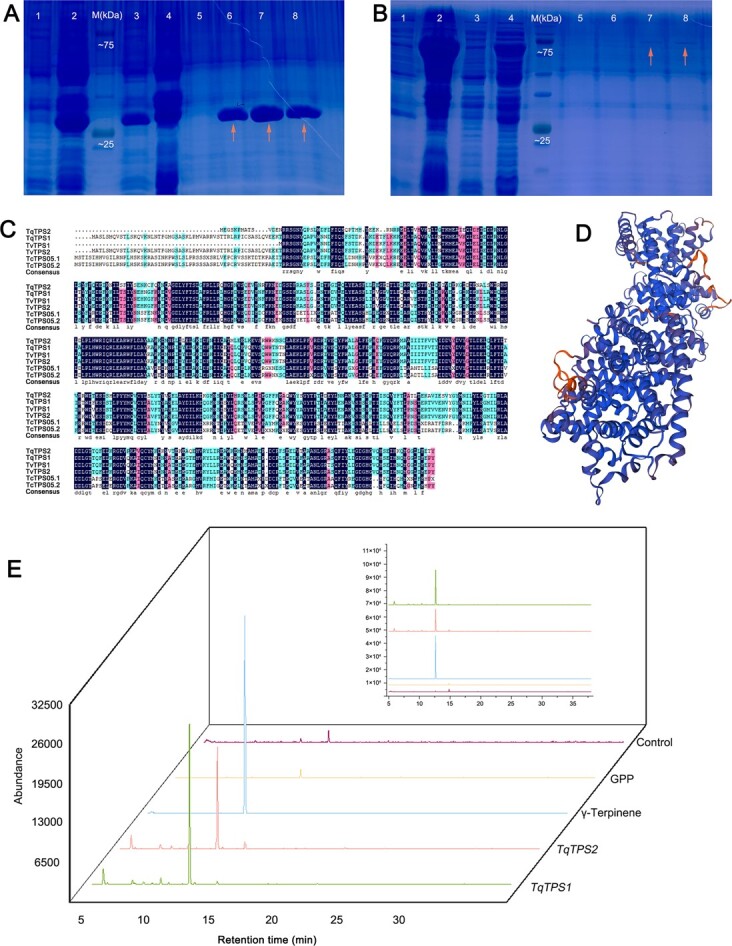
Protein expression, sequence alignment, structure, and *in vitro* enzymatic analysis of γ-terpinene synthase *TqTPS2* (*Tq13G005250.1*) from *T. quinquecostatus*. (A) Protein expression results for the control vector without γ-terpene synthase. 1, protein products after 0 hours of induction; 2, protein products after 12 hours of induction; 3, supernatant of purified protein; 4, sediment of purified protein; 5–8, eluant of purified protein (orange arrows indicate the eluant containing the target protein). (B) Protein expression results for the vector with *TqTPS2*. 1, supernatant of purified protein; 2, sediment of purified protein; 3, protein products after 0 hours of induction; 4, protein products after 12 hours of induction; 5–8, eluant of purified protein (arrows indicate the eluant containing the target protein). (C) Amino acid sequence alignment of TPS proteins from *T. quinquecostatus*, *T. caespititius*, and *T. vulgaris*. (D) 3D structure of the deduced TqTPS2 protein sequence. (E) *In vitro* enzymatic analysis of *TqTPS2* using GPP as a substrate. Enzymatic products of *TqTPS1* (green line) [22], enzymatic products of *TqTPS2* (red line), products of authentic standard γ-terpinene (blue line), products of GPP (yellow line), and products of pGEX-4 T (control vector without terpene synthase insert) (purple line).

The complete sequence of *Tq13G005250.1* (here referred to as *TqTPS2*) was cloned into pGEX-4T and expressed in the *Escherichia coli* BL21 strain, using an empty vector as a control (Fig. 12A and B). The sequence similarity between *TqTP2* and *TqTPS1* was 68.68% (Fig. 12C); however, they shared several conserved amino acid residues, including the highly conserved aspartate-rich motif DDxxD, the metal cofactor binding motif NSE/DTE, and the double arginine motif RRX8W. DDxxD binds to the divalent metal ion cofactor in the process of reaction [25], and RRX8W is indispensable to enzymatic activity and catalyzes the cyclization of monoterpenes [26]. The molecular mass of the protein encoded by *TqTPS2* was ~59 kDa. The 3D structure of the deduced *TqTPS2* protein is shown in Fig. 12D. *TqTPS2* was successfully induced in the supernatant and purified as a homogeneous soluble protein. The product peaks were identified by comparing mass spectra with the National Institute of Standards and Technology (NIST) library. The results indicated that *TqTPS2* can catalyze geranyl diphosphate (GPP) to form γ-terpinene (Fig. 12E). It was the second TPS verified by an enzyme activity assay *in vitro* in thyme.

## Discussion

*Thymus* includes more than 300 species with valuable aromatic and medicinal properties that are widely distributed worldwide, including many wild thymes in China [1, 2]. Thyme has a beautiful Chinese name, ‘${\includegraphics{\bwartpath uhac262fx3}}$’ (Bailixiang), in reference to its strong, pleasant fragrance, said to be detectable from 100 miles away. CNT species are widely used not only in folk medicine but also as a seasoning for food or as forage grass for livestock to improve the taste of meat [22]. We collected a large number of CNTs and ETs cultivated year-round in Beijing, China. These thymes showed interesting phenotypic variation. Some species were creeping-type thymes, like grasses, in which the aboveground part dies in the winter; other species were erect-type thymes, like little trees, with leaves falling off in the winter in China. European erect-type thymes are tall and widely cultivated for application in the food, pharmaceutical, and cosmetic industries [3]. Chinese creeping-type thymes also have valuable aromatic and medicinal properties. To facilitate mechanized harvesting in agricultural production and breed new varieties, we hybridized European erect-type thyme and Chinese creeping-type thyme. The chromosome-level genome of *T. quinquecostatus* (CNT39, an important wild thyme species in China) contains genes encoding the monoterpenes thymol and carvacrol, providing a resource for analyses of important horticultural traits [22]. In this study, we combined phenotypic data (e.g. growth form, terpenoid type and content, and glandular trichome density) with multiomics data and population genetic analyses, to evaluate lignin, terpenoid, and glandular trichome evolution.

We identified some CNTs, such as *T. mongolicus* (CNT08 and CNT11) and *T. quinquecostatus* var. *asiaticus* (CNT16), with high terpene contents, suggesting that these accessions can be used for agricultural production and functional assays (Fig. 2A). The 52 thymes formed three clusters in terms of leaf volatile terpenoid compositions, one including taxa rich in camphor, germacrene-D, and 1,8-cineol; another including taxa rich in *p*-cymene, γ-terpinene, thymol, and carvacrol; and another cluster including taxa rich in α-terpinyl acetate and α-terpineol (Fig. 2B and C). The second cluster contained the largest number of thyme species (i.e. 40 thymes). The glandular trichome density on leaves was 5.61–31.46 per mm^2^, and the area ranged from 5.63 to 35.25 mm^2^ (Fig. 3B and Supplementary Data Table S3). CNT04, CNT26, ET02, ET07, CNT09, CNT17, and CNT06 had high glandular trichome densities (>24.92 per mm^2^); CNT10, CNT18, CNT19, ET11, ET12, ET09, and ET08 had low glandular trichome densities (<12.73 per mm^2^). This variation provides a basis for studies of the mechanism underlying glandular trichome development, including QTL analyses of glandular trichomes by the hybridization of *F*_2_ and other populations. We found that CNTs clustered into three independent groups by a population genetic analysis, and these groups were consistent with the morphology-based classical taxonomy (Fig. 4). Of note, our findings suggested that *T. mongolicus* is a wild ancient thyme. The genetic diversity of cultivated accessions from Europe was higher than that of CNTs, different from results for some crops, like rice [27], but consistent with results for other plant taxa, such as tea [28]. The genetic diversity of cultivated thymes in Europe was higher than that of CNTs, which may be partially explained by ongoing introgression of CNTs into erect-type ET populations during long-term cultivation [28–30]. Evidence for selective sweeps was identified in dozens of genes, many of which were functionally related to the lignin content, terpenoid composition, and glandular trichome density. Further studies with extensive sampling of CNT and ET accessions are needed to trace their origins and confirm that these genes underwent selection during domestication.

The growth form of thyme is strongly linked to the lignin content in the stem. Lignin is a heterogeneous polymer, including *p*-hydroxyphenyl lignin, syringyl-lignin, and guaiacyl-lignin [31]. Our results indicated that most precursors resulted in a larger amount of caffeic acid in *T. vulgaris* ‘Elsbeth’ than in *T. quinquecostatus*, which may result in higher levels of downstream products, like ferulic acid, caffeyl aldehyde, and coniferyl alcohol, in *T. vulgaris* ‘Elsbeth’ than in *T. quinquecostatus* (Fig. 6). The erect-type trait of thyme may be closely related to the lignin content in the stem. COMT plays an indispensable role in lignin biosynthesis [8–10]. Our results indicate that levels of lignin biosynthesis are higher in erect-type *T. vulgaris* ‘Elsbeth’ than in creeping-type *T. quinquecostatus*. Terpenes are one of the main classes of plant-specialized multiple secondary metabolites, among which monoterpenes and sesquiterpenes are used in medicines and fragrances and function in plant defense. Terpene compounds are abundant in thyme [32, 33]. Interestingly, terpene compounds are generally synthesized and stored in an epidermal secretory organ, the glandular trichomes [34]. The glandular trichome-related genes identified in this study may affect the glandular trichome density and thereby the terpene content. By a weighted gene coexpression network analysis, we identified various regulatory genes associated with the three key horticultural traits, including *PER/LAC*, *TPS*, and *HD-ZIP*. These genes included the γ-terpinene synthase *TqTPS2* (*Tq13G005250.1*) of *T. quinquecostatus* (CNT39).

In short, we identified and classified 39 CNT accessions, including 11 species and 2 varieties. These CNT resources are expected to serve as a model for studies of the lignin content, terpenoid composition and content, glandular trichome density, and other traits in basic and applied research. Using these CNT accessions and newly constructed hybrids, we will continue to explore the genomic properties (physical maps) of other species of *Thymus.* We will combine physical maps with genetic maps to deeply analyze these three important horticultural traits, providing new directions for molecular breeding and the improvement of horticultural traits.

## Materials and methods

### Plant materials

Native *Thymus* species in China were investigated using the *Flora of China* [2] and local flora. Based on ethnobotanical information, 39 CNT accessions were collected directly from their natural habitats (Fig. 1) in China in 2018–19. For identification, herbarium specimens were examined at the Institute of Botany, Chinese Academy of Sciences (IB-CAS). Thirteen ET accessions were also used for reference and comparative analyses (Supplementary Data Fig. S1). All 39 CNT and 13 ET species were grown at IB-CAS, Beijing, China. Fresh leaves were taken and placed in liquid nitrogen as soon as possible. Before DNA extraction, the samples were stored at −80°C.

### Library construction and population-based resequencing

Genomic DNA of all thyme accessions was extracted using a DNA extraction kit. Libraries were constructed according to the manufacturer’s standard protocols (Illumina, San Diego, CA, USA). Then, 150-bp paired-end reads were generated using the Illumina NovaSeq 6000 platform. The sequencing depth for each individual was >7-fold (Supplementary Data Table S6).

### SNP and InDel variant detection and annotation

SNPs and InDels were detected using the GATK package [35]. Based on the localization of clean reads in the *T. quinquecostatus* reference genome (CNT39) [22], Sambamba (v0.7.1) [36] was used to filter redundant reads. Then, the GATK HaplotypeCaller (local haplotype assembly) algorithm was used for the detection of SNPs and InDels. Variant Call Format (gVCF) files for all accessions were generated for each sample, and population joint-genotyping was performed. After filtering, the final mutation site set was obtained. The generated variants were annotated using SnpEff based on the gene annotation of the *T. quinquecostatus* reference genome [37]. The polymorphic genes were searched against the GO [38], COG [39], KEGG [40], and other functional databases using BLAST [41], and annotations were obtained for functional enrichment analyses.

### Phylogenetic, admixture, population diversity, and linkage disequilibrium analyses

On the basis of the neighbor-joining method and the Kimura two-parameter model, we constructed a phylogenetic tree by MEGA X [42], and branch support was evaluated with 1000 bootstrap replicates. Admixture [43] was used to analyze population structure, setting the number of subgroups (*K*-value) to 1–10 for clustering. EIGENSOFT (v0.7.1) [44] was used for a PCA based on SNP datasets. The relationships between two individuals in a natural population were evaluated using GCTA [45]. To estimate LD patterns between different thyme groups, *R*^2^ was computed, and results were plotted using plink2 [46].

### Putative selective sweep analysis

*F*_ST_ and θπ were estimated to identify signatures of selection. *F*_ST_ and θπ values were identified as candidate outliers under strong selective sweeps and intersecting regions from the top 5% or 1% of different thyme populations. All tests were performed with a 100-kb sliding window and a 10-kb window step using PopGenome [47]. Candidate genes under selection (*P* < .05) were annotated genes in these retained regions. The candidate genes were evaluated by GO, COG, and KEGG enrichment analyses (false discovery rate <.05, *P* < .05).

### Phylogenetic analysis of genes encoding COMT, TPS, HD-ZIP, and MYB

A literature search of genes encoding COMT, TPS, HD-ZIP, and MYB was performed (Supplementary Data Tables S18–S21). Then, the corresponding amino acid sequences were obtained from NCBI [22]. COMT*-*, TPS-, HD-ZIP-, and MYB-encoding genes were authenticated by Fgenesh [48] and the MAKER-P pipeline [49]. Genes were predicted in the genome of *T. quinquecostatus* (CNT39) using hmmscan [50], and NLR-parser [51] was used for gene prediction in *T. quinquecostatus*, *A. thaliana*, *A. annua*, *N. tabacum*, *S. miltiorrhiza*, *S. baicalensis*, *P. virgatum*, and *V. vinifera*. Based on the SNP dataset, phylogenetic trees were constructed with the neighbor-joining method using FastTree (v2) [52] and visualized using ITOL (https://itol.embl.de/).

### Coexpression networks of genes related to lignin biosynthesis, terpenoid biosynthesis, and glandular trichome formation in thyme

Our previously published raw RNA-seq data for root, stem, leaf, flower, flower bud, and glandular trichome of ET02 and CNT39 [22] were filtered using Trimmomatic [53]. The clean reads were mapped onto predicted coding sequences in the genome using Bowtie (version 2.0) [54]. We used RSEM (version 1.3.2) (https://github.com/deweylab/RSEM) to calculate fragments per kilobase of exon per million fragments mapped (FPKM) [55]. Thresholds for DEG detection were *P* < .05 and log|fold change| > 2. Coexpression networks were generated by WGCNA v1.51 using all DEGs [56]. The networks including hub genes were visualized using Cytoscape v3.0.0.

### Identification of terpene constituents in leaves by headspace solid-phase microextraction

Terpene constituents of 52 thyme leaf samples were identified in three biological replicates following a previously reported method [22] by headspace solid-phase microextraction (HS-SPME). Briefly, 250 mg of leaf powder and 20 μl of 3-octanol (1 mg/ml) was placed into a headspace vial. We used crimp-top caps with TFE-silicone headspace septa to seal the vials quickly. Each vial was then incubated at 40°C for 0.5 hour. Then, to absorb the volatiles, the headspace of each vial was exposed to 100 μm polydimethylsiloxane-coated fiber for 0.5 h. All volatile organic compounds attracted to the fiber were analyzed by GC–MS [57]. The compound was identified by the comparison of mass spectra and retention times (RTs) with known compounds in the NIST v14.0 MS database and previously reported data [58]. Retention indices (RIs) were calculated using the following equation: RI = 100Z + 100[RT(*x*) − RT(*z*)]/[RT(*z* + 1) − RT(*z*)], where RT(*x*), RT(*z*), and RT(*z* + 1) indicate the composition and retention times for carbon numbers Z and Z + 1 of the reference alkane.

### Observation of glandular trichomes

Glandular trichomes were observed and counted using a stereomicroscope, fluorescence microscope, and scanning electron microscope following a previously reported method [22]. The density of glandular trichomes on the abaxial and adaxial leaf surfaces was calculated as the ratio of the number of glandular trichomes to the leaf area.

### Determination of lignin biosynthesis intermediate metabolites in the stem

Two freeze-dried stems were smashed using a mixer mill (MM 400, Retsch). Then, 0.1 g of powder was extracted overnight at 4°C with 0.6 ml of 70% aqueous methanol. After the extracts were absorbed and filtered by centrifugation, the samples were analyzed using an UPLC-ESI-MS/MS system. On the basis of the MWDB database and the public metabolite information database, the primary and secondary spectral data were analyzed qualitatively. For the analysis of metabolite structure, we referred to MassBand, KNApSAcK, HMDB, MoTo DB, and METLIN. We used triple quadrupole MS to quantify metabolites by multiple reaction monitoring [59].

### Hybrid breeding design

ET as the female parent was crossed with CNT, with the growth form, terpenoid content, and glandular trichome density as the main breeding goals. Different combinations were constructed according to the target trait in 2020. Finally, we selected six *F*_1_ populations for further analysis, including *T. longicaulis* (ET10) × *T. quinquecostatus* (CNT39), *T. longicaulis* (ET10) × *T. mongolicus* (CNT22), *T. longicaulis* (ET10) × *T. quinquecostatus* var. *przewalskii* (CNT24), *T. longicaulis* (ET10) × *T. vulgaris* ‘Fragrantissimus’ (ET04), *T. vulgaris* ‘Elsbeth’ (ET02) × *T. quinquecostatus* (CNT39), and *T. vulgaris* ‘Elsbeth’ (ET02) × *T. quinquecostatus* (CNT22).

### RNA extraction and cDNA synthesis

Different tissues from root, stem, leaf, flower, flower bud, and glandular trichome of *T. vulgaris* ‘Elsbeth’ and *T. quinquecostatus* were collected and stored at −80°C. Total RNA was extracted from the frozen leaves using an RNA Extraction Kit. A NanoDrop spectrophotometer was used to determine the concentration and a Bioanalyzer 2100 (Agilent Technologies) to analyze RNA integrity. Then, the HiScript Reverse Transcriptase Kit (Vazyme, Nanjing, China) was used to synthesize cDNA [60].

### Gene expression using qRT–PCR

To analyze the expression patterns of 12 vital genes in the three pathways, primers were designed using Primer3 (http://primer3.ut.ee) (Supplementary Data Table S22). Then, qRT–PCR was carried out on a CFX96 instrument (Bio-Rad, Hercules, CA, USA) using the SsoFast EvaGreen Supermix Kit in a 20-μl reaction volume containing 1 μl of template cDNA, 0.8 μl of right and left primer, 10 μl of 2× T5 Fast qRT–PCR Mix, 0.4 μl of 50× ROX Reference Dye II (TSINGKE, Beijing, China), and 7 μl of nuclease-free water. The qRT–PCR protocol included 40 cycles of 95°C for 30 seconds, 95°C for 10 seconds, and 58°C for 30 seconds, along with a melting curve analysis. The relative quantification was performed using the internal reference genes *18S rRNA* and *β-actin*. Data were presented using the 2^-ΔΔCT^ method based on the normalization of transcript levels. All analyses were performed in triplicate [61].

### Heterologous expression of *TqTPS2*

To identify the function of *TqTPS2*, the coding sequence was amplified with primers and successfully cloned into the expression vector pGEX 4T. Meanwhile, an empty vector (as a control) and vectors ligated with *TqTPS2* were transformed into *E. coli* strain BL21 (DE3). Isopropyl-β-d-thiogalactopyranoside (IPTG) (0.5 mM) was used to induce the expression of recombinant *TqTPS2* at 18°C for 12 hours in an incubator with constant shaking. Subsequently we centrifuged the cells, added phosphate-buffered saline to resuspend them, and broke them down by sonication. Crude proteins were then applied to glutathione beads. GPP was used as the substrate to be catalyzed by the purified protein, and the final product was detected using GC–MS.

### Statistical analysis

Statistical analysis was performed using IBM SPSS Statistics for Windows, version 19.0 (Armonk, NY, USA). Thyme accessions were compared by one-way ANOVA, followed by Duncan’s multiple range test at the 5% probability level (*P* ≤ .05).

## Acknowledgements

This work was supported by the Strategic Priority Research Program of the Chinese Academy of Sciences (grant no. XDA23080603). We thank Xiuping Xu and Ronghua Liang from the Plant Science Facility of the Institute of Botany, Chinese Academy of Science, for their excellent technical assistance in scanning electron microscopy and fluorescence microscopy. We thank Yan Zhu from the Plant Science Facility of the Institute of Botany, Chinese Academy of Science, for her excellent technical assistance in MS analysis.

## Author contributions

M.Y.S. and Y.N.Z. performed the experiments, analyzed the data, and wrote the manuscript; H.T.B. and G.F.S. collected samples and analyzed the data; J.Z.Z. collected samples and designed the research; L.S. was involved in research design and revising the manuscript. All authors read and approved the manuscript.

## Data availability

The raw sequence data for resequencing have been deposited in NCBI under project accession no. PRJNA690675. All supplementary figures and tables are provided in the Supplementary Data files.

## Conflict of interest

The authors declare no competing financial interests.

## Supplementary data

Supplementary data are available at *Horticulture Research* online.

## Supplementary Material

Web_Material_uhac262
