# Supplementary material for: Population diversity analyses provide insights into key horticultural traits of Chinese native thymes
Source: Hortic Res. 2022 Aug 2;10(2):uhac262. doi: 10.1093/hr/uhac262 (PMC9907056; doi:10.1093/hr/uhac262)
Supplement: Web_Material_uhac262 [file web_material_uhac262.zip › Supplemental Figure.pdf]

Supplementary Figures

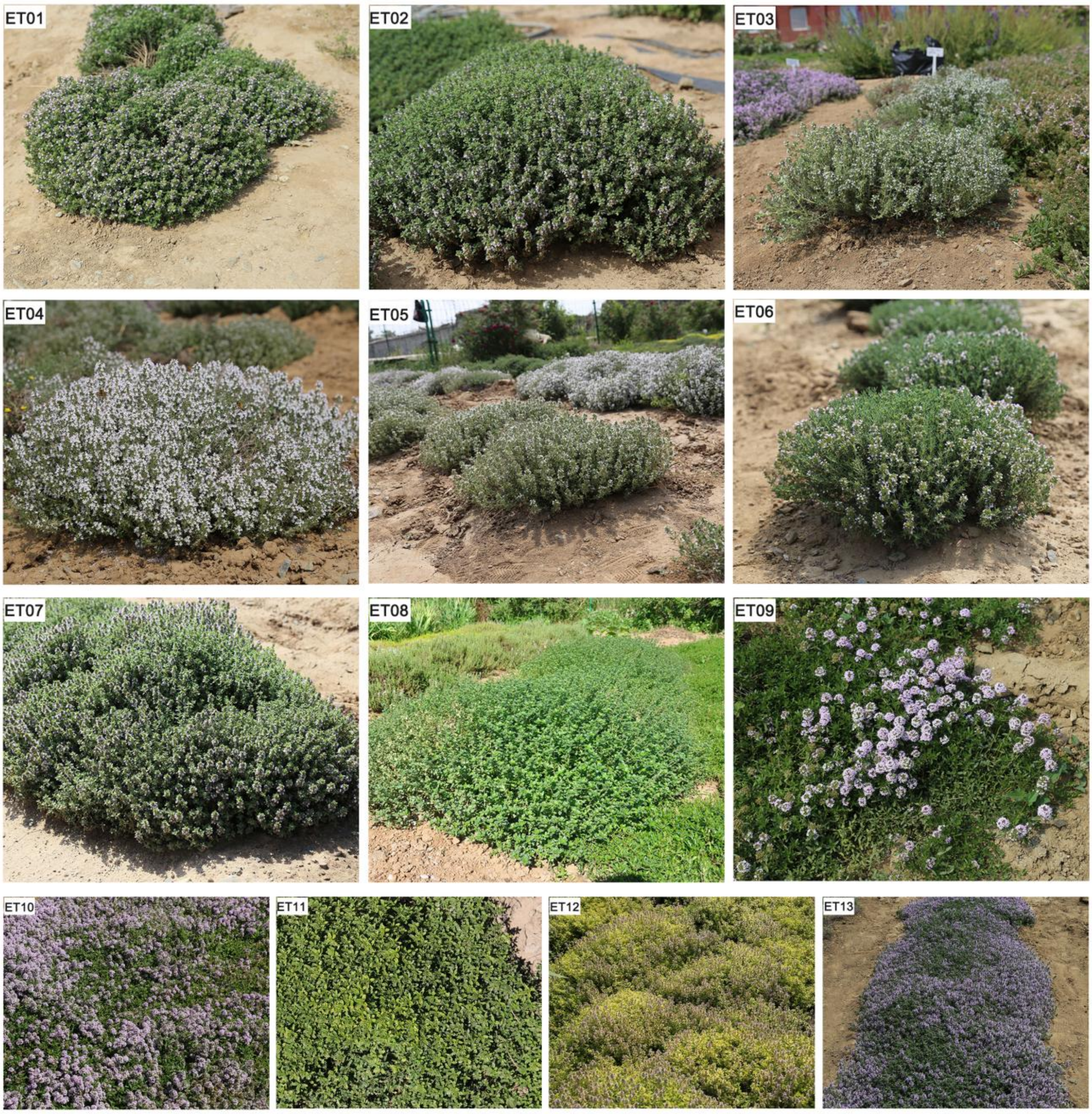

Fig. S1. Images of European thymes (ET). ET01, *T. vulgaris* 'Compactus'; ET02, *T. vulgari*s 'Elsbeth'; ET03, *T. vulgaris* 'Fleur provenule'; ET04, *T. vulgaris* 'Fragrantissimus'; ET05, *T. vulgaris* 'Pink selection'; ET06, *T. thracicus*; ET07, *T. rotundifolia*; ET08, *T. comosus*; ET09, *T. guberlinesis*; ET10, *T. longicaulis*; ET11, *T. pulegioidess* 'Golden Dwarf'; ET12, *T. serpyllum* 'Aureus'; ET13, *T. praecox* Opiz subsp. *polytrichus* (A. Kern. ex Borbs) *Jalas*. ET01–ET07 are erect-type thymes (ETE), and ET08–ET13 are creeping type thymes (ETC) .

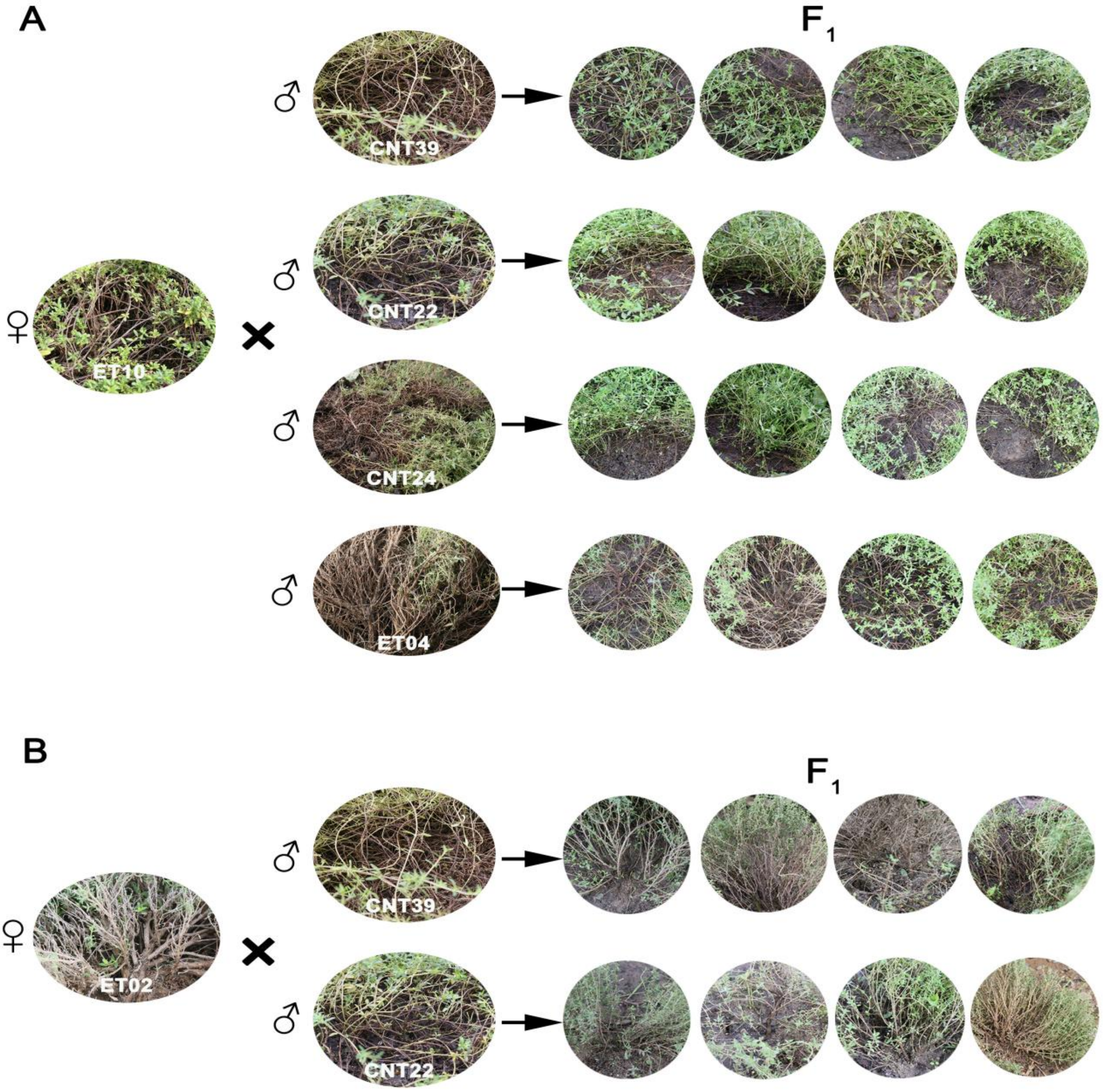

Fig. S2. Images of the stem in six parental populations and F_1 lines. (A) Stem images of ET10 (female parent) and CNT39/CNT22/CNT24/ET04 (male parent), and their F_1 lines. ET10, *T. longicaulis*; CNT39, *T. quinquecostatus*; CNT22, *T. mongolicus*; CNT24, *T. quinquecostatus* var. *przewalskii*; ET04, *T. vulgaris* 'Fragrantissimus'. ET10 (female parent) and CNT39/CNT22/CNT24 (male parent) are creeping-type and their F_1 lines are creeping-type. ET10 (female parent) is creeping-type, ET04 (male parent) is erect-type, and their F_1 lines are creeping-type or semi-creeping-type. **(B)** Stem images of ET02 (female parent) and CNT39/CNT22 (male parent), and their F_1 lines. ET02, *T. vulgaris* 'Elsbeth'; CNT39, *T. quinquecostatus*; CNT22, *T. mongolicus*. ET02 (female parent) is erect-type, CNT39/CNT22 (male parent) are creeping-type, and their F_1 lines are erect-type or semi-erect-type. The population parents were all two-year old cutting seedlings. F_1 lines' seeds were planted on October 5, 2020. The stems were photographed on August 20, 2021.

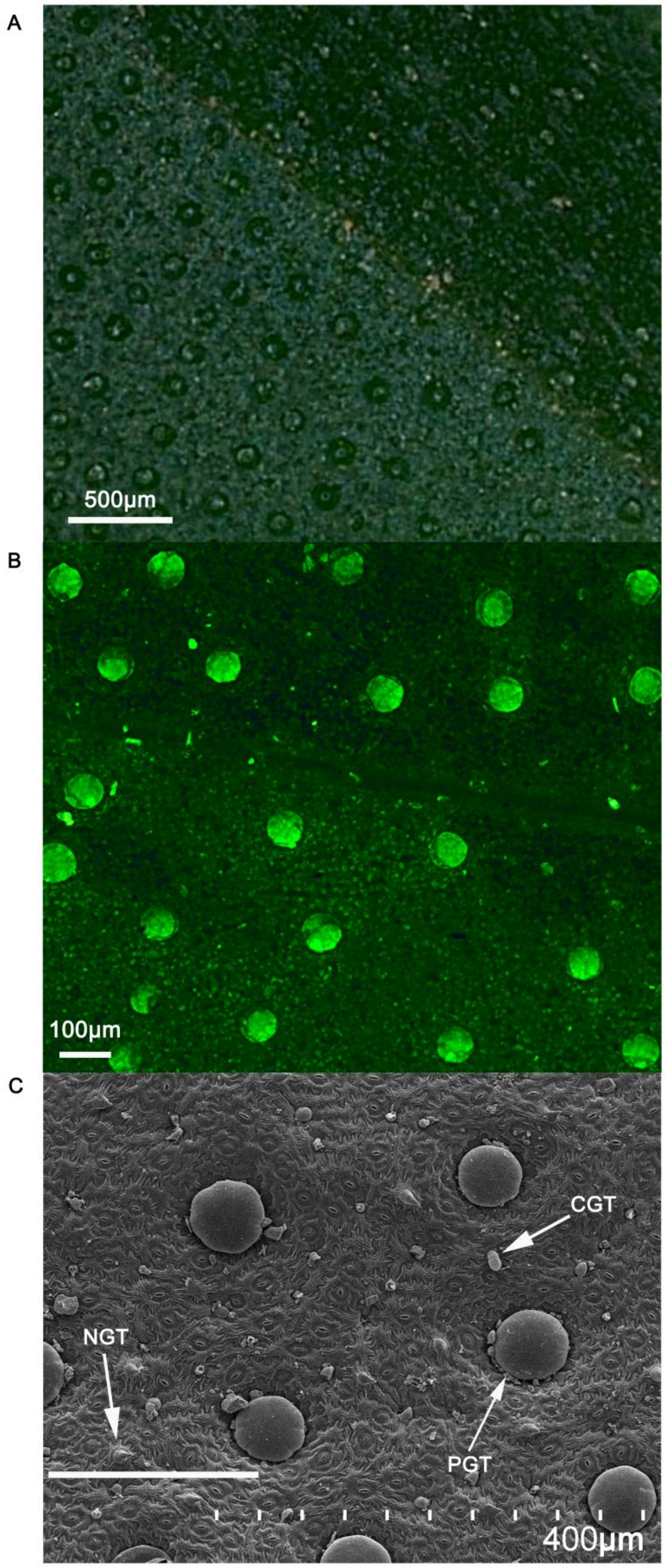

Fig S3. The morphology of glandular trichome in thyme. (A) The image was observed by using stereomicroscope. **(B)** The image was observed through capturing the green autofluorescence of glandular trichome by using fluorescence microscopy. **(C)** The image showed scanning electron microscope (SEM) analysis of the leaf surface of thyme.

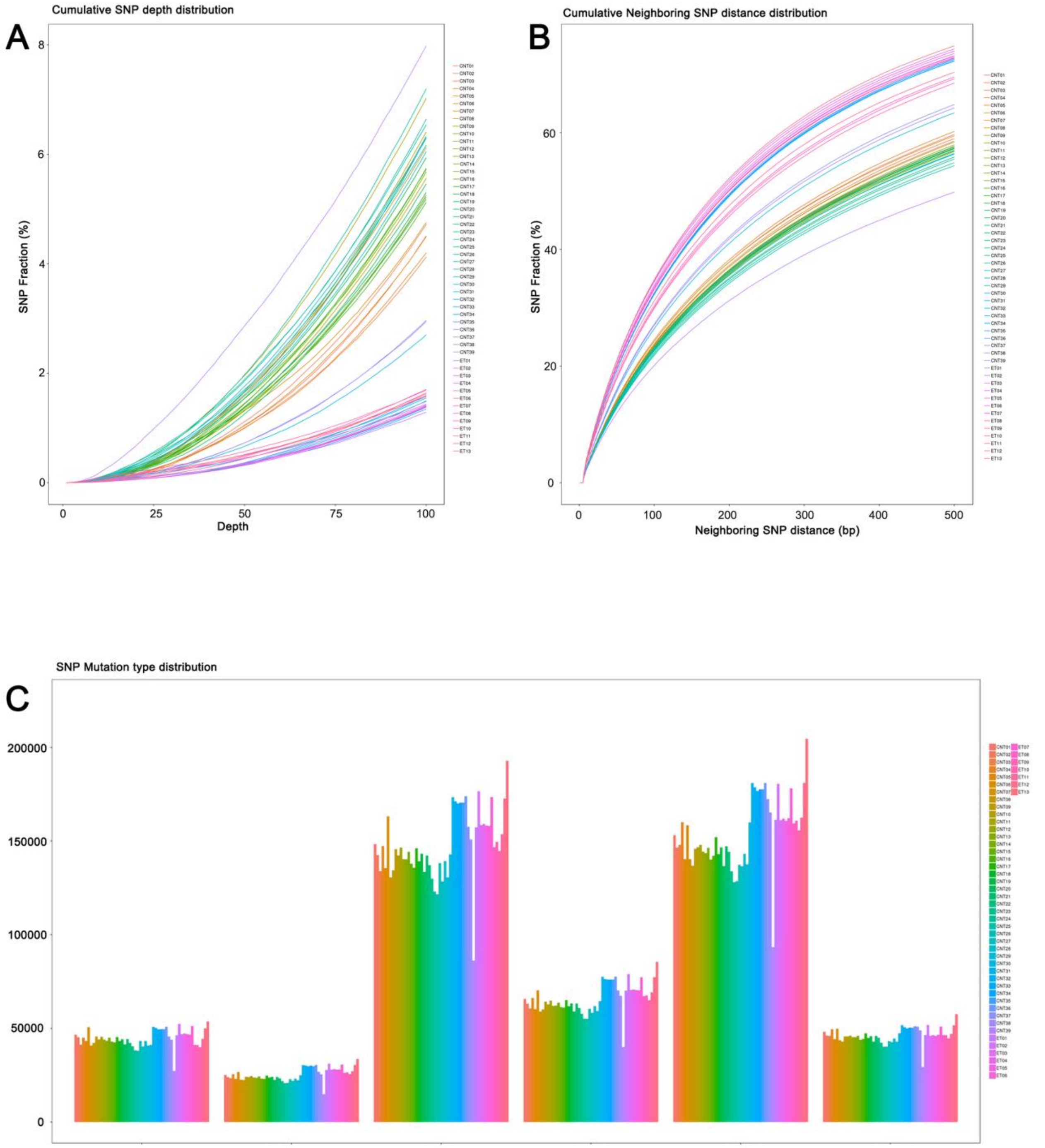

Fig. S4. Genome-wide SNP distribution for 52 thyme accessions. (A) Cumulative SNP depth distribution of 52 thyme accessions. **(B)** Cumulative neighboring SNP distance distribution of 52 thyme accessions. **(C)** Six SNP mutation types distribution of 52 thyme accessions.

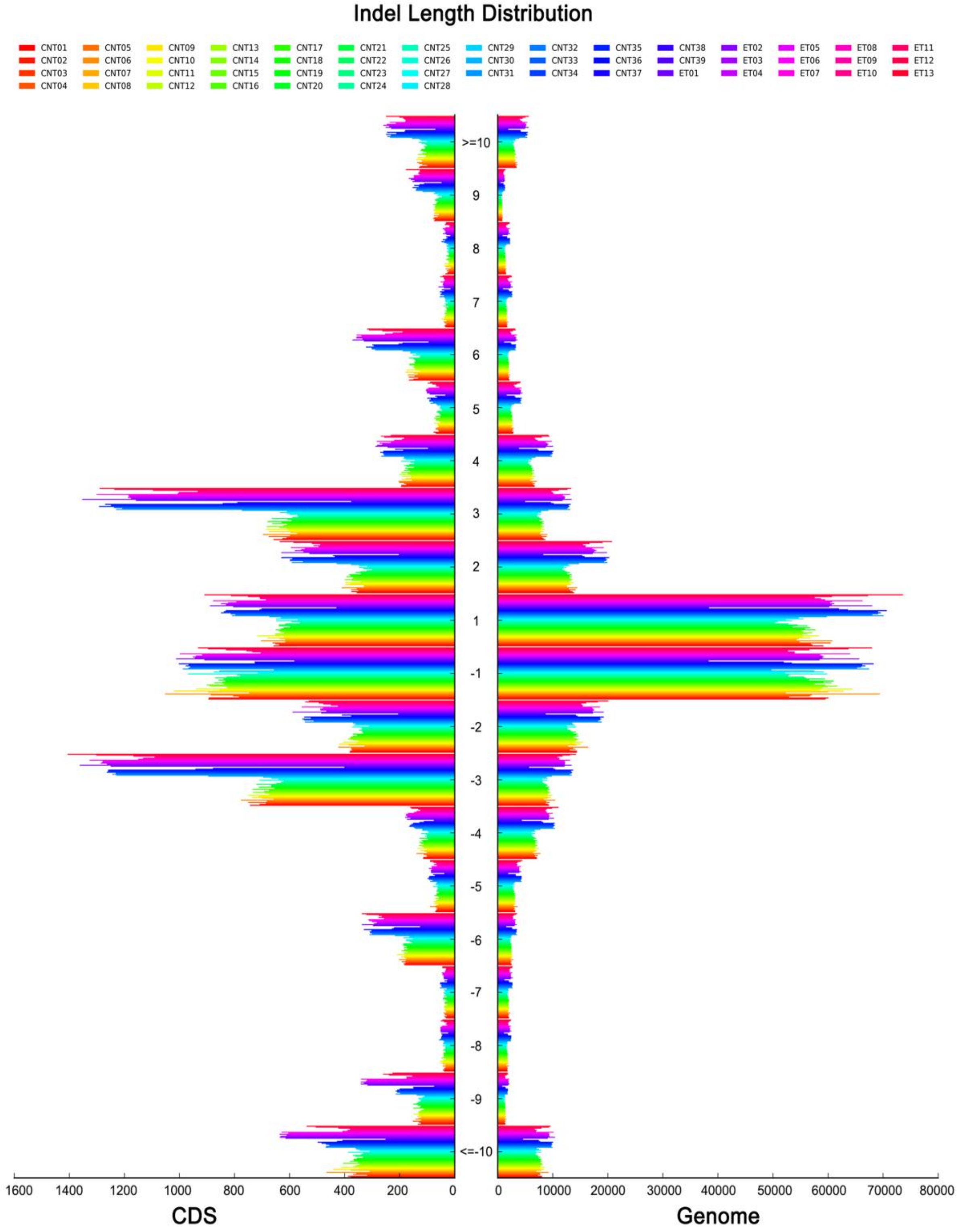

Fig. S5. Genome-wide InDel length distribution for 52 thyme accessions.

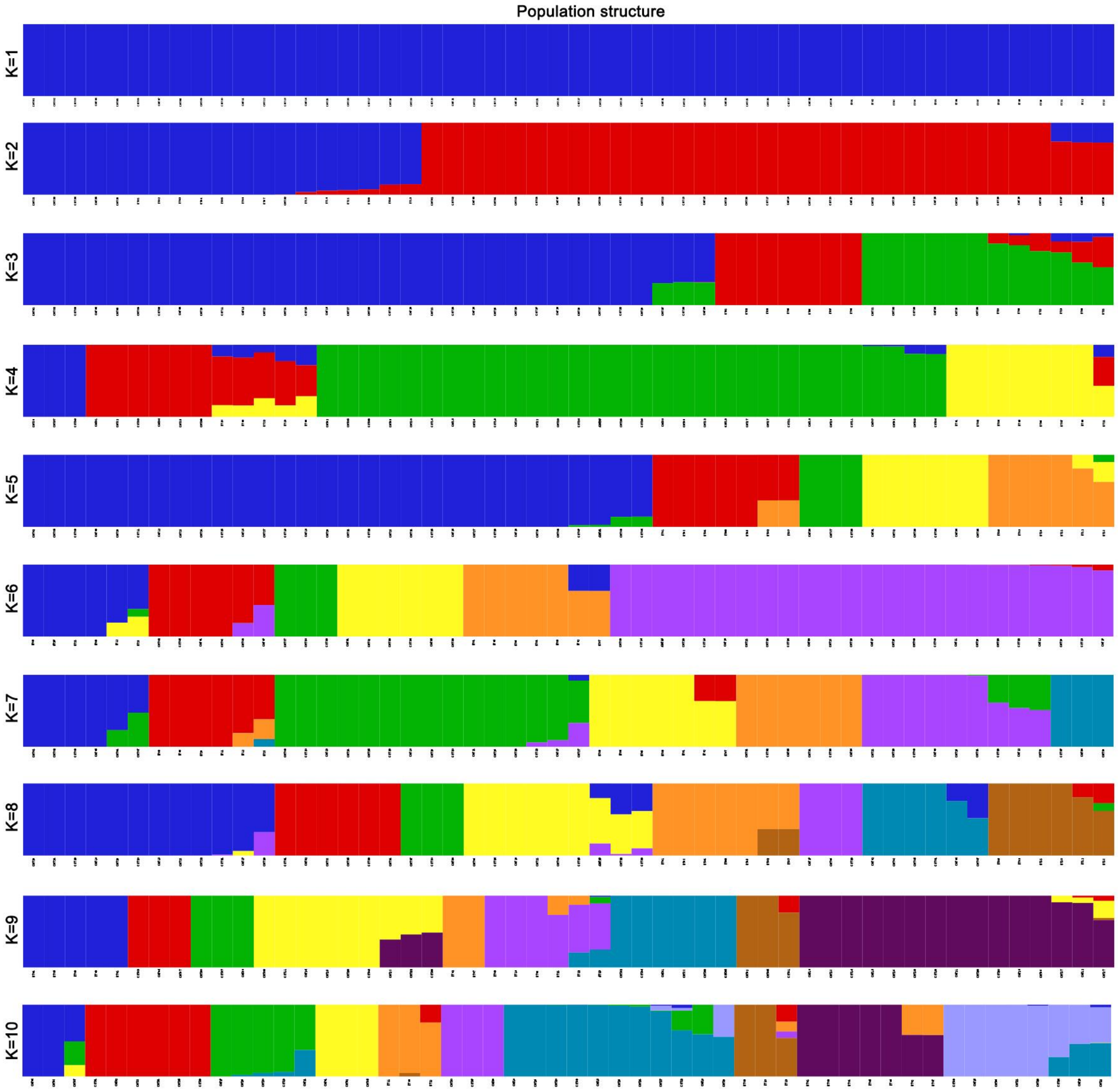

Fig. S6. Structure of the thyme populations (K value of 1–10).

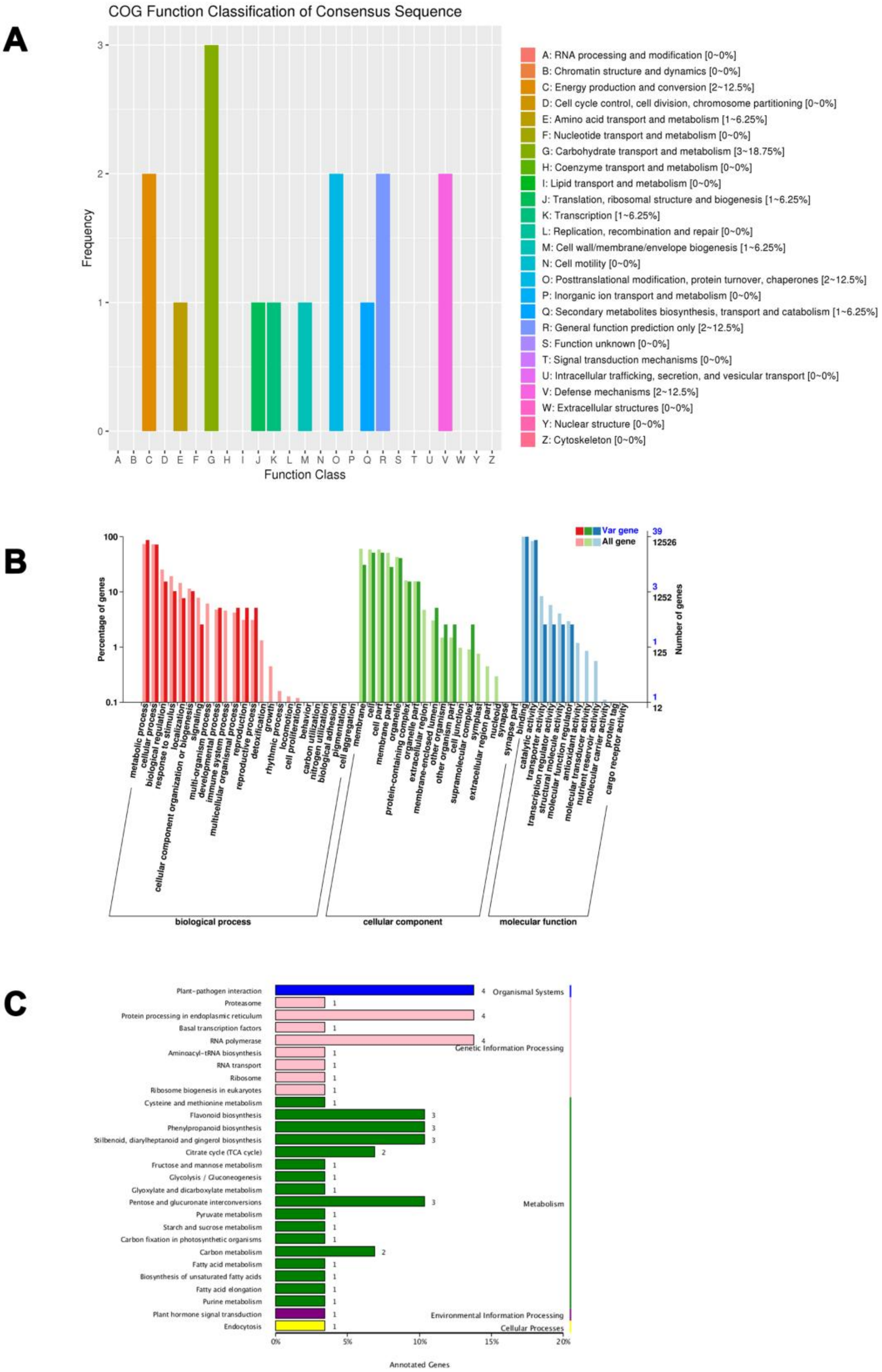

Fig. S7. COG, GO, and KEGG annotation of genes under selection identified by *Fst* and θπ between CNTC and ETC populations. (A) COG annotation of the selective genes identified by Fst and θπ between CNTC and ETC populations. **(B)** GO annotation of the selective genes identified by Fst and θπ between CNTC and ETC populations. **(C)** KEGG annotation of the selective genes identified by Fst and θπ between CNTC and ETC populations.

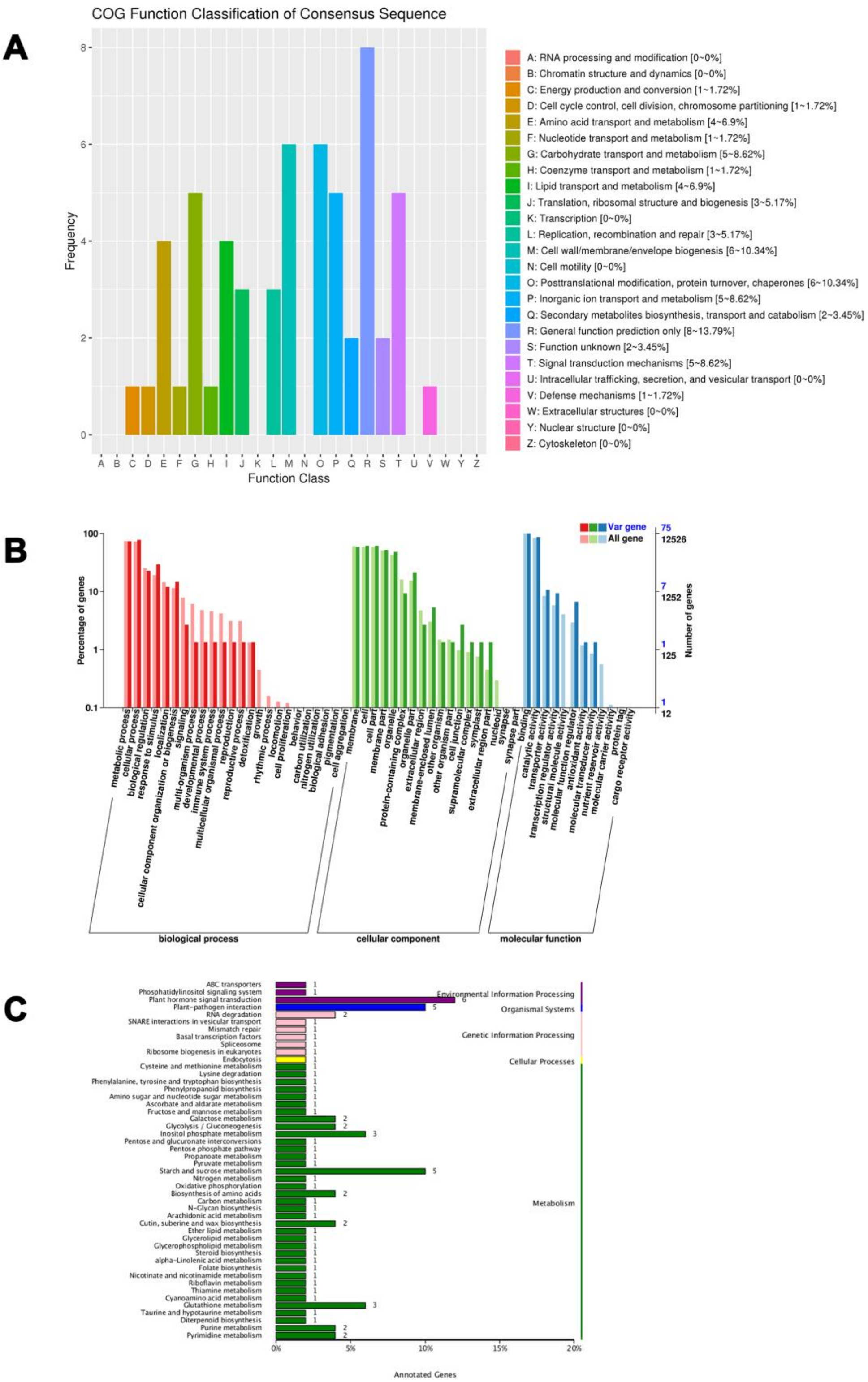

Fig. S8. COG, GO, and KEGG annotation of genes under selection identified by Fst and θπ between CNTC and ETE populations. (A) COG annotation of the selective genes identified by Fst and θπ between CNTC and ETE populations. **(B)** GO annotation of the selective genes identified by Fst and θπ between CNTC and ETE populations. **(C)** KEGG annotation of the selective genes identified by Fst and θπ between CNTC and ETE populations.

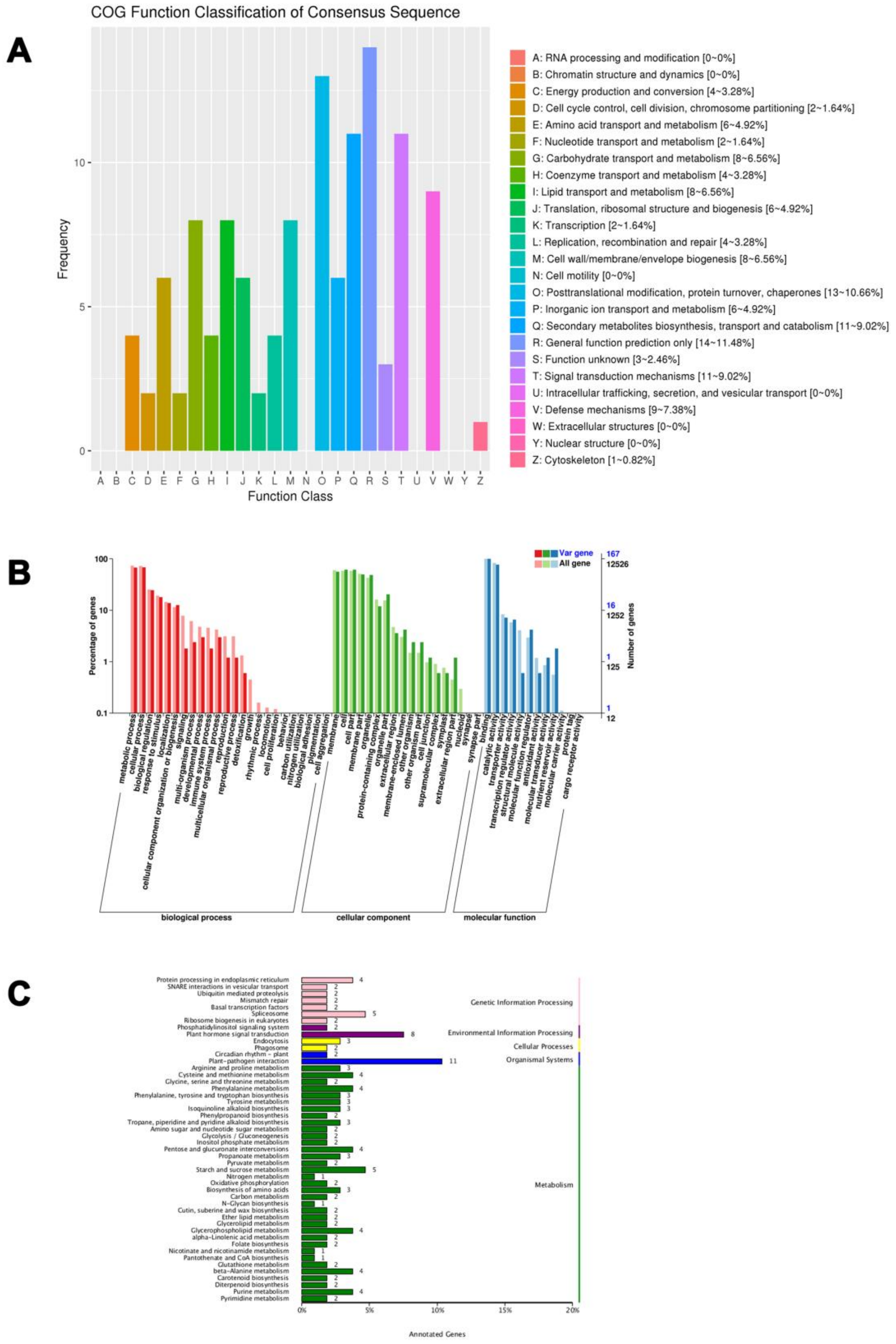

Fig. S9. COG, GO, and KEGG annotation of genes under selection identified by Fst and θπ between ETC and ETE populations. **(A)** COG annotation of the selective genes identified by Fst and θπ between ETC and ETE populations. **(B)** GO annotation of the selective genes identified by Fst and θπ between ETC and ETE populations. **(C)** KEGG annotation of the selective genes identified by Fst and θπ between ETC and ETE populations.

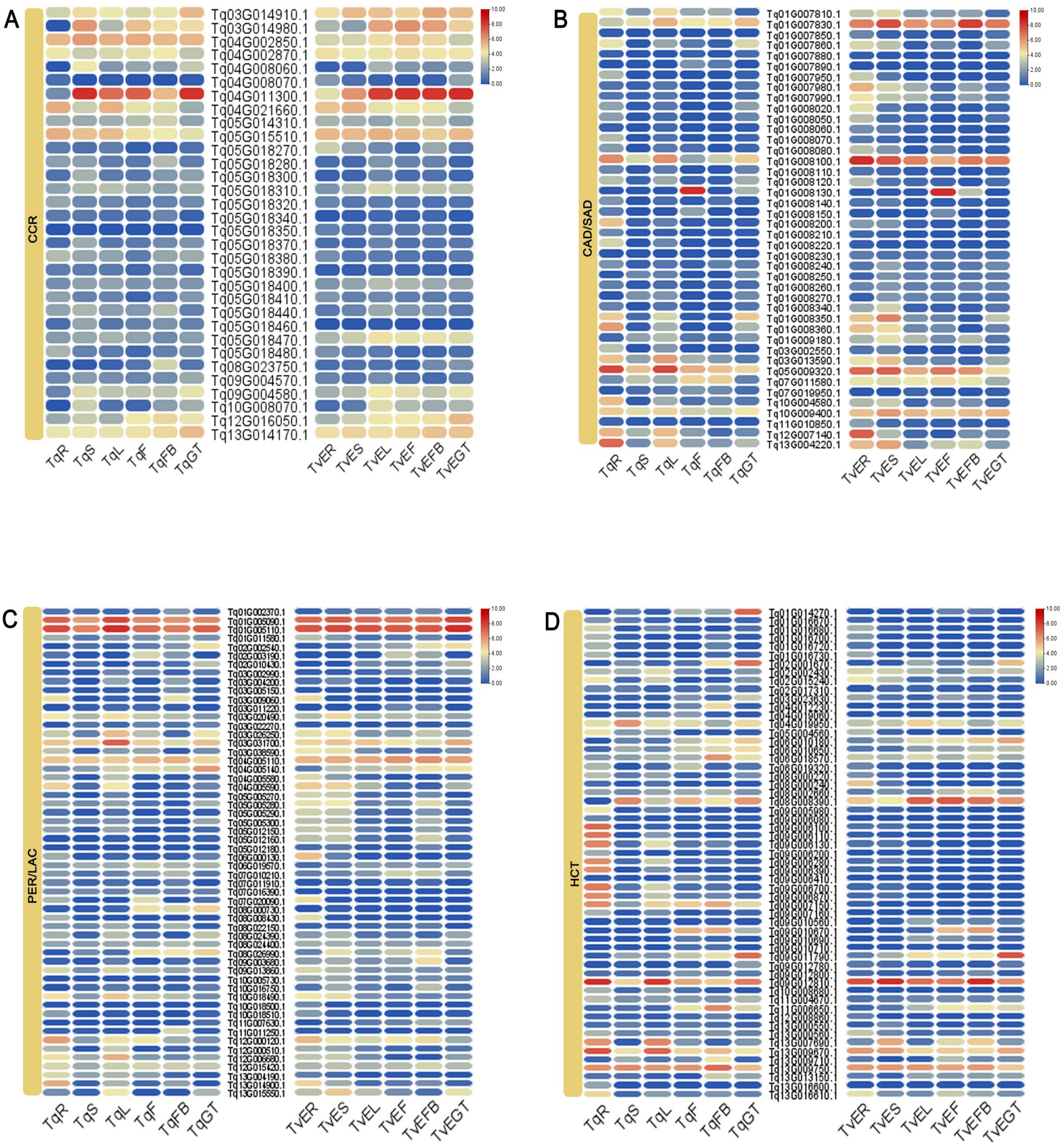

Fig. S10. Expression heatmap of *CCRs*, *CAD/SADs*, *PER/LACs*, and *HCTs* involved in lignin biosynthesis. (A) Expression heatmap of *CCRs* in lignin biosynthesis. **(B)** Expression heatmap of *CAD/SADs* in lignin biosynthesis. **(C)** Expression heatmap of *PER/LACs* in lignin biosynthesis. **(D)** Expression heatmap of *HCTs* in lignin biosynthesis. The bar at the right top marked the normalized FPKM values for both in *T. quinquecostatus* and *T. vulgaris* 'Elsbeth' (red denoted high expression and blue indicated low expression, expression data were Z-score standardized to -3 to 3 per gene.). All of expression genes showing minimum two-fold changes at $p < 0.05$ (Student's two tailed t-test). TqR, *T. quinquecostatus* root; TqS, *T. quinquecostatus* stem; TqL, *T. quinquecostatus* leaf; TqF, *T. quinquecostatus* flower; TqFB, *T. quinquecostatus* flower at the bud stage; TqGT, *T. quinquecostatus* glandular trichome; TvER, *T. vulgaris* 'Elsbeth' root; TvES, *T. vulgaris* 'Elsbeth' stem; TvEL, *T. vulgaris* 'Elsbeth' leaf; TvEF, *T. vulgaris* 'Elsbeth' flower; TvEFB, *T. vulgaris* 'Elsbeth' flower at the bud stage; TvEGT, *T. vulgaris* 'Elsbeth' glandular trichome.
